# Supplementary material for: Improving the 3D representation of plant architecture and parameterization efficiency of functional–structural tree models using terrestrial LiDAR data
Source: AoB Plants. 2024 Dec 24;17(2):plae071. doi: 10.1093/aobpla/plae071 (PMC11826235; doi:10.1093/aobpla/plae071)
Supplement: plae071_suppl_Supplementary_Materials [file plae071_suppl_supplementary_materials.pdf]

Title: Improving the 3D Representation of Plant Architecture and Parameterization Efficiency of Functional-Structural Tree Models using Terrestrial LiDAR Data

Main Author: Vera Bekkers
Affiliation: Earth Systems and Global Change Group, Wageningen University, The Netherlands
Country: the Netherlands

Co-author: Jochem Evers
Affiliation: Center for Crop System Analysis, Wageningen University, The Netherlands
Country: the Netherlands

Co-author: Alvaro Lau*
Affiliation: Laboratory of Geo-Information Science and Remote Sensing, Wageningen University, The Netherlands
Country: Peru
*corresponding author

Abstract

Background and Aims: Functional-Structural Plant (FSP) models are useful tools for understanding plant functioning and how plants react to their environment. Developing tree FSP models is data-intensive and measuring tree architecture using conventional measurement tools is a laborious process. Light Detection and Ranging (LiDAR) could be an alternative non-destructive method to obtain structural information about tree architecture. This research investigated how terrestrial LiDAR (TLS)-derived tree traits could be used in the design and parameterization of tree FSP models.

Methods: A systematic literature search was performed to create an overview of tree parameters needed for FSP model development. The resulting structural parameters were compared to LiDAR literature to get an overview of the possibilities and limitations. Furthermore, a tropical tree and Scots pine FSP model were selected and parametrized with TLS-derived parameters. Quantitative Structural Models (QSM) were used to derive the parameters and a total of 37 TLS-scanned tropical trees and 10 Scots pines were included in the analysis.

Key Results: Ninety papers on FSP tree models were screened and 8 papers fulfilled all the selection criteria. From these papers, 50 structural parameters used for FSP model development were identified, from which 28 parameters were found to be derivable from LiDAR. The TLS-derived parameters were compared to measurements, and the accuracy was variable. It was found that branch angle could be used as model input, but internode length was unsuitable. Outputs of the FSP models with TLS-derived branch angle differed from the FSP model outcomes with default branch angle.

Conclusions: Results showed that it is possible to use TLS for FSP model inputs, although with caution as this has implications for the model variable outputs. In the future, LiDAR could help improve efficiency in building new FSP models, increase the accuracy of

existing models, add metrics for optimization, and open new possibilities to explore previously unobtainable plant traits.

Keywords: LiDAR, Terrestrial Laser Scanner, Functional-Structural Plant Models, Quantitative Structural Models, Tree Architecture.

1 Introduction

Plant growth models have played an important part in gaining insights into plant functioning and improving crop yield productivity. The first plant growth models were developed at the beginning of the 20th century (Blackman, 1919), and since then there have been exponential advancements. One of the important drivers of progression is the rapid developments in computer processing power and accessibility. However, plant growth models are limited in that they cannot take into account the variation of individual plants and their interactions with the environment.

To help solve this limitation Functional-Structural Plant (FSP) models were introduced. Functional-Structural Plant models are a type of plant growth model that incorporates both the physiological processes and the 3D structure of a plant (Sievänen et al., 2000). These models can be used to study plants on different scales, from cell level to whole plant communities (DeJong et al., 2011). The incorporation of both structure and functioning allows FSP models to account for the feedback between them and helps to better understand plant growth in relation to its environmental conditions (Vos et al., 2010). This interaction makes FSP models unique and allows the study of growth in heterogeneous plant canopies, like mixed crop species (Gaudio et al., 2019; Evers et al., 2019) and to a lesser extent, mixed forest stands (Bongers, 2020). Findings from research using FSP models help in the development of management strategies in agriculture, horticulture, and forestry (DeJong et al., 2011; Boudon et al., 2020), and can for example be used to study practices like agroforestry (Barbault et al., 2024). Additionally, they can be used for fire management in forests (Parsons et al., 2011), to better understand the behaviour of trees in response to extreme climate events (Taugourdeau and Barczi, 2013) and render more realistic synthetic trees in 3D (Crimaldi et al., 2021).

The incorporation of both detailed functional processes and the 3D architecture makes FSP models more complex than other plant growth models. This is especially valid for

modelling forest stands, because of their large structure and long lifespan. In the past, the main constraints for building and using FSP models for forests were related to the high computational intensity and data needs (Sievänen et al., 2000). The first constraint has been partly solved with the exponential developments in computer processing power (O'Sullivan et al., 2021), and some mixed forest models have been developed (Hemmerling et al., 2008; Petter et al., 2021). However, the large amount of data needed for model development and validation are still a relevant problem today (Louarn and Song, 2020). An important component of FSP model development is thus to develop techniques to efficiently acquire 3D structures of plants (Sievänen et al., 2014).

Different instruments can be used to acquire structural plant measurements. Conventionally used manual measurement tools are rulers, callipers, compasses, angle finders and hypsometers (Van der Heijden et al., 2007). More detailed measurements are extracted from 3D data, acquired using conventially used tools like 3D digitizers (Surový et al., 2011), which can often be used outdoors, and 3D reconstructions from images or lasers, which are captured using equipment which are often fixed indoors (Dornbusch et al., 2007; Paulus et al., 2014). These techniques work well for small plant structures (e.g. annual crops) which can be moved and be measured indoors, but for large plant structures (e.g. trees), collecting data with the use of these techniques is often impractical and laborious to acquire. For example, digitizing two complete tree crowns in 3D with a Polhemus FASTRAK took Surový et al. (2011) 14 days, as each 3D coordinate had to be individually captured using a pointer. Additionally, the high architectural variation between trees compared to annual plants makes full tree-scale validation more difficult, and as a result tree models are often validated qualitatively (Grisaf et al., 2022).

Light Detection and Ranging (LiDAR) could be an alternative sampling method for obtaining structural information of trees for FSP modelling compared to conventionally used

methods. Light Detection and Ranging is a non-destructive and active remote sensing method, which can capture the 3D architecture of trees in high detail (Newnham et al., 2015). The technique works by sending out laser beam pulses and recording the time it takes after hitting an object for the laser to travel back (in ‘time-of-flight’ systems) (Baltsavias, 1999) or by measuring the phase shift signal between a continuous outgoing one and its reflected counterpart (in ‘phase shift’ systems) (Disney, 2019). The amount of laser beam pulses can reach up to millions per scan position (Brede et al, 2017; Wilkes et al., 2017). The recorded time of each laser can then be used to calculate the distance between the scanner and the object and together with the recorded GPS location a highly detailed 3D scan is constructed, also called a point cloud data (PCD) (Mallet and Bretar, 2009).

1.1 LiDAR for Collecting Structural Tree Information

There are several devices available for obtaining LiDAR scans of trees. The most widely used are Terrestrial Laser Scanner (TLS), Airborne Laser Scanner (ALS), and more recently Mobile Laser Scanner (MLS). The techniques for acquiring and processing LiDAR have progressed substantially in the last years, making it possible to retrieve valuable information from LiDAR data. Diameter at breast height (DBH) and height are accurately derivable from LiDAR data, although the accuracy are highly dependent on understory growth because of occlusion (Brede et al., 2017; Liu et al., 2018). Additionally, it is possible to extract more complex tree characteristics from PCD such as, crown diameter (Popescu et al., 2003), leaf area index (Zheng et al., 2012), plant area index (Calders et al., 2015b) and branch details (Lau et al., 2018). It is also possible to calculate tree attributes like biomass through allometric models (Zolkos et al., 2013) and plant scaling with metabolic scaling exponents (Lau et al., 2019b). LiDAR is also useful for better understanding tree functioning (Malhi et al., 2018; Dorji et al., 2021) and measuring the effect of forest management strategies (Georgi

et al., 2018). There are many examples of the use of LiDAR for capturing tree details and for more information the review of Calders et al. (2020) can be read.

One of the methods to reconstruct the LiDAR scanned trees is through single tree reconstruction modelling. The accuracy to which the 3D tree models can be constructed varies in levels of detail of retrievable parameters and complexity to acquire (Liang et al., 2016). For example, leaves and higher-order branches require a high level of model detail compared to height and DBH. There are different methods for reconstructing 3D structural tree models, each with advantages depending on the scan quality and goal of the tree model (Boudon et al., 2014; Bournez et al., 2017). Quantitative methods are often used to reconstruct 3D structural tree models, the two most common methods being skeletonization (Côté et al., 2009, Côté et al. 2011) and Quantitative Structural Models (QSMs) (Raumonen et al., 2013; Delagrange et al., 2014). Both methods translate the PCD as an input into an architectural tree model from which architectural information can be derived. Skeletonization converts the PCD into a series of segments which are geometrically and topologically connected which results in the representation of a tree (Côté et al., 2009). TreeQSM is a popular 3D tree model developed by Raumonen et al. (2013) which uses fitted cylinders of the PCD to derive several tree attributes, such as tree height, branch angle, branch length, branch diameters, among others. Advantages are that the method is able to reconstruct multiple trees with high accuracy (Raumonen et al., 2015).

These 3D structural tree models can be used to interpret remote sensing data through Radiative Transfer Model simulations (Disney et al., 2006; Calders et al., 2018) or to help enhance the quality of the PCD (Côté et al., 2011). Additionally, it is possible to extract characteristics from the 3D structural tree models which can be used for forest inventories (Liang et al., 2016; Aijazi et al., 2017) or model inputs (e.g. wind damage modelling (Jackson et al., 2019)).

The ability to scan large plots of trees in a short time and extract tree characteristics non-destructively are important benefits that make LiDAR an increasingly viable data source. However, depending on the LiDAR methods used (TLS, ALS, or MLS) there are tradeoffs in time efficiency and details. Additionally, the highly detailed scans done by TLS (e.g. 3 mm accuracy recorded by Brede et al. (2017)) allow for deriving more complex tree characteristics not measurable by conventional methods (Liang et al., 2016). Research about deriving tree characteristics from LiDAR has started to mature, and the next step will be to look at other research fields which can benefit from this data (Disney, 2019). Tree FSP modelling could be the next research field that can benefit from LiDAR data, increasing efficiency in building the models and improving them through optimization or validation, compared to conventional methods.

1.2 LiDAR and FSP Modeling

O'Sullivan et al. (2021) defines four possibilities for tree pointclouds from TLS data to be used in FSP models.

1. Direct use of 3D tree models (QSMs) derived from TLS for simulations of physiology and environment.
2. Provide validation data for testing the FSP models.
3. Acquire time-series of tree pointclouds to get data about the growth dynamics.
4. Using pointcloud time-series data for optimization of parameters.

Several of these possibilities have been explored in recent research. Sievänen et al. (2018) demonstrated the use of TLS data for optimizing a shoot-based FSP model. TLS-scanned pine trees were divided into internodes and used to validate the predictions of the

model. These different outcomes were then used to decide the best-fitting method for crown development. This study shows the potential of TLS for component selection, but due to the small sampling size, they disclosed that the results should be viewed as preliminary. Another study done by Beyer et al. (2017) focused on using TLS data to compare the 3D output of a beech FSP model. The TLS data was used for visual comparison and validation using relative leaf densities. Perez et al. (2018) used TLS data to derive indicators for evaluating the performance of a 3D architectural oil palm model. Several basic indicators were derived in combination with hemispherical photographs, such as plant height, width, volume and gap fraction. Bailey (2019) integrated an automatic LiDAR processing plugin to its modeling framework called Helios. The leaf inclination distribution was derived from TLS data and used as parameter input for a case study to simulate Canopies of *Prunus dulcis*. Finally, Potapov et al. (2017) used TLS data for parameter optimization to simulate virtual trees. The TLS scans were reconstructed in a QSM from which structural features are extracted and used as inputs in an optimization algorithm.

The above-mentioned studies demonstrate individual cases that showcase how TLS data can be used for optimization, model component selection, parameter input and validating the structural output of a model. However, the more general question regarding the extent to which TLS-derived tree characteristics could be used for FSP model development has not been examined. Additionally, a fifth option could be explored, apart from the four mentioned by O’Sullivan et al. (2021), which is to use TLS-derived parameters from a single time frame for FSP model inputs.

1.3 Research Objectives and Research Questions

TLS-derived tree parameters have the capability to be a valuable source of information for FSP models but have largely been unexplored until now. This research aimed to investigate how TLS-derived tree traits could be used for tree FSP models by providing model inputs.

First, an overview of FSP model development needs was created and the possibilities of LiDAR to be an alternative data source were determined. Next, two FSP models were selected based on several criteria and the structural inputs were analysed to see which could be replaced with TLS-derived parameters from our literature review. Then, the suitable parameters were derived from TLS data and the accuracy was analysed. These parameters were then used as inputs for the selected models and the effect on variable outputs were assessed. This research can be an example case and findings could be used for future research and encourage more exploration in the potential of TLS data for tree FSP model development.

2 Data and Methods

A systematic literature review was performed to create an overview of FSP model structural data needs and if these tree parameters could be derived from LiDAR data (Section 2.1). For the next part, structural data derived from LiDAR were used as inputs in the FSP models and the outputs were compared with the default settings. For comparison two FSP models were selected and introduced in Section 2.2. Structural parameters were identified from the model inputs which could be derived from the LiDAR data. The LiDAR data used is described in Section 2.3. Preprocessing steps and Quantitative Structural Models (Raumonen et al., 2013) are described in Section 2.4 and derivation of TLS structural parameters for FSPM inputs are described in Section 2.5. The outputs of the QSM were assessed by comparing them to fieldwork observations and manual measurements from CloudCompare (Section 2.6). Finally, the procedure and output assessment of running the FSP models with TLS-derived inputs and models with default inputs are described in Section 2.7. For a schematic overview of the methods we refer to **Supplementary Information Fig. S1.**

2.1 Literature Review

A systematic literature search was performed to create a representation of the structural parameters used in FSP models. Different tree FSP models were identified, because of the variation in structural details needed for different types of FSP models. Parts of the Preferred Reporting Items for Systematic Reviews and Meta-Analyses (PRISMA) statement were followed to improve transparency and help structure the reporting of the process (Moher et al., 2009).

The following search engines were used for the literature search: Google Scholar, Web of Sciences and Scopus. To ensure that the FSP models would be focused on tree modelling the articles had to contain the word "tree". Additionally, the article had to contain

some variations of the word FSP model, like Functional-Structural Tree and Functional-Structural forest model or the corresponding abbreviations. The alternative term "virtual plants" was also included (Hanan, 1997). The following search term using Boolean functions was used on the 15th of June in 2024:

“tree AND ("Functional Structural Plant model" OR "Functional Structural forest model" OR "Functional Structural tree model" OR "FSP model" OR "virtual plant" OR FSPM OR FSTM OR FSFM)”

The hit results for the first 50 results were recorded for each of the three search engines. Duplicate records were removed and the abstracts of the remaining records were screened. Papers were included only if they met all of the following four criteria: be a published paper, the topic should be about FSP models, an original article that developed or improved an FSP model, and the FSP model was about a non-fruit bearing tree. The choice to not include fruit trees was to narrow the scope to make the process feasible within the given time frame. The papers that met the first inclusion criteria were retrieved and the methodology sections were skim-read to assess the eligibility. There were two inclusion criteria for the next selection round: the paper had to specify which parameters were used for the FSP model and manual field measurements should have been done by the authors. This last criterion was created as papers that use other databases are often not specific in how these field measurements were carried out and what exact parameters were retrieved. The review is thus not a complete overview of all parameters used in the selected papers but gives an indication of the field measurements that are done for FSP model development.

2.1.1 LiDAR for FSP Model Structural Parameters

The final selection of papers was then fully read, and the structural parameters that were measured for the tree FSP model development were identified. This includes all the data that was gathered for parametrization, optimization and validation.

An additional search was done for each parameter to find literature related to extracting structural tree information from LiDAR. In total, one paper for each parameter was selected. The search was performed between November 2021 and January 2022. The paper selection was performed by searching in Google Scholar, with the structural parameter together with the term LiDAR. More recent publications and papers with more citations were preferred for the LiDAR literature. The type of LiDAR equipment and accuracy of the estimation were recorded for the chosen papers. Each parameter was also compared with the output results of the QSM and deemed possible if the parameter could be retrieved from this.

2.2 FSP Model Selection

The decision was made to only focus on FSP models developed in GroIMP (Growth Grammar related Interactive Modelling Platform), because of available support for this software. GroIMP is a free Interactive modelling Platform created to develop, use and analyse the outcomes of FSP models (Hemmerling et al., 2008). Nine tree FSP models were identified from which two were selected for the analysis **[Supplementary Information Table S1]**. The choice was made based on the LiDAR tree genus and species data availability and the documentation.

The first selected FSP model is a tropical tree and forest model (Petter et al., 2021) and the second is a LIGNUM model adapted for a Scots pine tree *(Pinus sylvestris)* (Smoleňová et al., 2013). Each model was assessed to identify potential structural parameters which could be changed to LiDAR-derived data. A limitation was that the LiDAR datasets

did not contain any temporal information. Nonetheless, the FSP models could still benefit from data that is collected from a particular time point by focusing on parameters that do not change over time.

The tropical FSP model from Petter et al. (2021) uses an ecophysiological approach to look at the effect of leaf traits on the growth patterns of single trees and forest stands. For the analysis, only the individual tree model was analysed. To change the input parameters for this model a pass file with parameter values can be modified. The parameters listed in this file were assessed to identify model inputs that could be derived from the QSM, using the results from section 2.1. Four structural parameters were identified: branch angle for first and second branch order and internode length of the trunk and first-order branches.

The LIGNUM model of a Scots pine was originally described in Perttunen et al. (1998) and Sievänen et al. (2008) and was adapted by Smoleňová et al. (2013) to run in GroIMP. One individual Scots pine tree is modelled which grows in a forest and is competing for light. Parameters can be changed through a pass file or directly modified in the model. All parameters specified in the pass file and parameters used in the model itself were assessed using the results from the literature review. Two structural parameters were identified that could be derived from LiDAR in the module L-systems for pine trees: branch angle for first and second branch order.

2.3 LiDAR Data

Two LiDAR datasets of segmented trees were used for this study based, and details about the species and field measurements are described in Table 1. The first LiDAR dataset contains segmented Scots pine trees from a plot in Loobos, the Netherlands, scanned in September 2011 with a RIEGL VZ-400 terrestrial laser scanner. The forest stands age is approximately 100 years old and has a composition of predominantly Scots pine with sparse understory.

This study site has repeated measurements available and field observations were used that were collected in March 2012. Further detail of the LiDAR data acquisition can be found in Vaccari et al. (2013) and the segmentation steps were according to Lau et al. (2019a).

The second data set of segmented tropical trees used for this study was collected during a field campaign in the East Berbice-Corentyne Region of Guyana. The scanned plot was situated in a mixed forest dominated by evergreen trees. The scanning was done from January to February 2017 using a RIEGL VZ-400. Additionally, field observations were available of the height and DBH which were measured at the same time. Further details about the segmentation, scanning setup and sampling plan can be found in Lau et al. (2019a).

2.4 Preprocessing

It was decided to use the QSM developed by Raumonen et al. (2013) to acquire TLS-derived tree parameters. This method was chosen as the output of the QSM gives a wide range of relevant and detailed information about the tree architectures from which the selected tree parameters could be derived. The QSM requires only the woody part of the tree as input, so the classification algorithm of D. Wang et al. (2020) was used to select the woody parts and discard the rest. Published pre-processed pipelines from Lau et al. (2019a) were followed in MATLAB (R2019b) to run the QSM and classification algorithm.

The QSM and leaf separation algorithm was run using an Intel Xeon W-2133 running at 3.60 GHz with 128 GB RAM. MATLAB had 6 workers during the calculations. Additional scripts (available upon request)9were written in Python (3.8) for analyzing and visualising the results.

2.4.1 Woody Component Extraction

The algorithm of D. Wang et al. (2020) requires one parameter to be specified which is the feature similarity called N_z*thres*. A sensitivity analysis was performed to find the best performing threshold using qualitative visual scoring **[Supplementary Information Table S2].** We analysed the results from each performing threshold ranging from 0.025 to 0.225 with a 0.025 step **[Fig. S2-S3]**. The best performing N_z*thres* for tropical trees was found to be 0.1 for the smallest and largest DBH class trees and 0.125 for the remaining trees. For the Scots pine, the N_z*thres*value 0.15 was found to retain the most wood detail and wrongly classify the least amount of soft components.

After inspecting the classification results it was decided to apply manual corrections to the LiDAR data. This included manual re-classifications of wrongly classified branches and removing wrongly classified foliage and ghosting effect (Fig. 1). This step was performed using the segmentation tool in the program CloudCompare (v2.11.3 (Anoia)). The effect of manually correcting the data on the QSM was further analysed to see if there are effects on the outputs of the QSM. All Scots pine trees were used for the analysis and one random tree was chosen for each DBH class for the tropical LiDAR data. Finally, a t-test was performed for the branch angle normal distribution to test if there are significant differences between the results of the QSM.

2.4.2 QSM Fitting

The method from Raumonen et al. (2013) uses a cover-set approach where surface patches are applied and connected step-wise over the PCD until they cover the whole tree surface. Cylinders of varying sizes can then be fitted using the patched tree surface. From the fitted cylinders, relevant information can be extracted about the tree branching structure. A complete list of outputs can be found in the TreeQSM documentation, section 1.7

(https://github.com/InverseTampere/TreeQSM/blob/master/Manual/TreeQSM_documentation.pdf).

The QSM results include three methods to calculate DBH, which were all tested to find the best-performing method. For both the Guyana and Loobos data set the *DBHqsm* method was found to have the highest accuracy, so for further analysis, this DBH calculation method was used. It was decided to also use an additional height estimation method which uses the original PCD with the foliage still included and takes the highest point and subtracts the lowest point from this (Calders et al., 2015a).

2.5 Deriving Structural Parameters for the FSP Models

The results from the QSM were modified to fit the structural parameter definitions used in the FSP models. The model inputs of branching angle for the first and second branch order for the Scots pine model and the second-order branch for the tropical tree model could directly be used from the QSM output. However, the branch angle of the first-order branch for the tropical tree model required the branch angle to be relative to the horizontal plane instead of being related from the trunk to the first-order branch. To correct for this the branch angle was calculated by taking the first-order branch angles and subtracting this from 90 degrees. The assumption made here was that the trunk grows straight up.

Internode length is defined by Petter et al. (2021) as the distance between two consecutive branches on the stem or parent branches. The internode length was calculated by finding the start coordinates of the first cylinder in a branch from the QSM output. The distance between the starting point of branches was calculated using Equation 1. With d being the distance, and x, y and z being the coordinates of the two branch starting locations.

$$(1) \qquad d = \sqrt{(x_2 - x_1)^2 + (y_2 - y_1)^2 + (z_2 - z_1)^2}$$

2.6 Accuracy Analysis

Field measurements from the Guyana and Loobos datasets were used to assess the accuracy of the height and DBH derived from the LiDAR data. Additionally, measured data of the branch angle was gathered directly from the PCD that served as input for the QSM. The first branch was taken of each tree, counting from the ground, for both first and second-order branches. The condition was that the branch had to have a length longer than 10 cm, due to erroneous modelling of the QSM and visually inspecting them.. Three points were visually picked in CloudCompare, one on the branching point and the other two along the branches with a length between 10-40 cm, depending on occlusion and changing shapes of the branch. The branch angle of the first branch order relative to the horizontal plane could not be measured this way as CloudCompare needs points present to measure the angle, so the same measurement method was used as the other branch angles. The measured branch angles were noted down together with the height of the beginning of the branch and the length of measurement.

The Root Mean Square Error (RMSE) (Equation 2), relative RMSE(%) (Equation 3) and R^2 (Equation 4) were calculated to assess the accuracy of the LiDAR estimated DBH, height and branch angle. yˆi the predicted value and yi is the measured. The relative RMSE(%) is calculated by taking the RMSE and dividing this by the mean of the observations (yi).

$$(2) \qquad RMSE = \sqrt{\sum_{i=1}^{n} (\frac{(\hat{y_i} - y_i)^2}{n})}$$

(3) $$RMSE(\%) = \frac{\sqrt{\sum_{i=1}^{n}\left(\frac{(\hat{y_\iota} - y_i)^2}{n}\right)}}{\bar{y_\iota}}$$

(4) $$R^2 = \frac{\sum_{i=1}^{n}(\hat{y_\iota} - \bar{y_\iota})^2}{\sum_{i=1}^{n}(y_i - \bar{y_\iota})^2}$$

2.7 FSP Model Running with TLS-Derived Inputs

The TLS-derived structural parameters were used as inputs for the FSP models and compared with the default FSP models (original values of the provided input file). The outputs were used to understand the sensitivity of the FSP models for these different parameter inputs. All models were run in GroIMP (V1.6). It was decided to average the maximum and minimum of the factor controlling the relationship between internode length and total annual length growth to avoid changes between runs in the tropical model.

Next, the structural parameters derived from TLS were used as inputs for the FSP models. No other parameters were changed as the focus was on using TLS-derived parameters. There were a total of 4 runs performed for each tree species: the median, 25th percentile, 75th percentile and a normal distribution function. For both branching angles and internode lengths, it was chosen to have the minimum and maximum range be the 25th and 75th percentile and take the median to remove the influence of outliers. The normal distribution was taken for all parameters to keep it the same for all. The input parameter was replaced directly in the code with the normal distribution function (normal(μ, σ)). This was done to make sure that for each new branch, a new value from the normal distribution was taken instead of one fixed value.

2.7.1 FSP Model Output Comparison

The selected FSP models were run the same amount of time steps as mentioned in the original papers, with each time step representing one year. The tropical tree model was run for 200 years and 40 years for the Scots pine model. Pictures were saved for each time step and used for visual comparison between the different runs. Additionally, structural outputs were saved for both models for each time step. The variables that were chosen to compare the tropical tree and Scots pine model outputs were: diameter (m), height (m), woody (Mg tropical tree model and kgC Scots pine) and leaf biomass (g tropical tree model and kgC Scots pine). Four additional variables were looked at for the tropical tree model: crown depth (m), crown area (m^2), height first branching (m) and light measured by apical meristems at each end of the branch and trunk (μmol m^2s^{-1}). The Scots pine also had four additional variables for comparison between the different model outcomes which were: root biomass (kgC), total segments (n), photosynthetic rate (kgC $year^{-1}$) and respiration (kgC $year^{-1}$).

3 Results

3.1 Literature Review

The literature search using the PRISMA method resulted in the identification of 150 records, with an additional 21 papers added manually (Fig. 2). From these initial records, 47 duplicates were removed and the remaining 124 were screened from which an additional 91 records were excluded as they did not meet the inclusion criteria. Most papers were excluded during the first criteria round because the modelled tree was a fruit-bearing species. The remaining 33 articles were then retrieved for the second criteria assessment. Two articles were excluded in this stage as retrieval was not possible due to accessibility issues. The remaining 31 studies were then fully read and 21 articles were excluded during this stage.

Most were excluded because they did not do manual field measurements, but referred to other literature for acquiring structural parameter data. A total of 10 studies met all criteria and were used for the literature review.

Overview LiDAR Possibilities for FSP Model Development

The 10 articles that came out of the systematic literature search were used to create an overview of FSP model structural parameter needs (Table 2). A total of 50 tree parameters were identified from the papers, ranging from the tree neighbourhood level to foliage details. Specific mentions of each parameter in the selected papers are described in **[Supplementary Information Table S2]**. Measured parameters for individual papers ranged from 4 (Buck-Sorlin et al., 2008; Kang et al., 2018) to 15 (Lu et al., 2011; F. Wang et al., 2011; Guo et al., 2012), and an average of 11 structural parameters were measured in the studies. The most often measured parameters among the papers were tree species (10), height (7), age (7), DBH (7), internode length (6), branch order (6) and branch angle (5). Parameters about details of neighbouring trees and foliage were mentioned in only a few papers.

From the 50 found parameters, 28 were found to be derivable from LiDAR data (Table 2). Accuracy was reported in different metrics among different papers which makes general intercomparison unviable. However, it was frequently reported that lower-order branches had lower accuracy than higher-order branches. Furthermore, 12 of the parameters can also be derived directly from the output of the QSM of Raumonen et al. (2013) and an additional 12 has the potentiality to be derived from the cylinder information. For example, chord length could be calculated by taking the locations of the first and last cylinder of a branch. The potentiality of LiDAR declines as the level of detail of the parameters increases. All parameters from the neighbourhood and tree level are found to be extracted directly or indirectly from PCD. The majority was found to be achieved for the whorls and branch

parameters and parameters on internode and foliage level were found to be feasible in less than half of the cases.

3.2 Terrestrial LiDAR Pointcloud Preprocessing

Thirty-seven tropical trees were selected from six tropical tree species. Additionally, ten Scots pine trees were used for deriving tree parameters. The process outputs of the preprocessing steps and QSM fitting are shown in Fig. 3. The average time spent manually correcting one tree was 45 minutes. Time spent on manually correcting the PCD depended on tree size, classification algorithm performance and complexity of the tree. Running the QSM for all manually corrected tropical trees took an average of 89 minutes and direct outputs of the LeWoS algorithm took 147 minutes. The Scots pine trees took an average of 13 minutes to run for all manually adjusted trees and an average of 49 minutes for non-adjusted trees.

3.2.1 Effect of Manual Corrections

QSM outputs that had manually corrected inputs differed from non-corrected QSM results (Table 3). Results showed low percentage changes for DBH, tree height, trunk volume and branch volume (0% - 3%). Larger differences were found for total volume, trunk length, branch length, number of branches, max branch order and total area (8 %-80 %). Percentage changes were similar between the tropical trees and Scots pine for the parameters with small changes. However, the Scots pine trees showed larger differences in total volume, trunk length, branch length and total area.

A tropical tree, a Mora gonggrijpii (ID: 80_14), was selected for visualization and statistical intercomparison between pre and post-manual cleaning (Fig. 4). The figure of the manually corrected tree shows less noise compared to the non corrected one, resulting in

better distinguishable branches. Looking at the quantitative results it becomes evident that the manually corrected tree resulted in fewer branches and a lower standard deviation and mean. Additionally, an independent t-test was done and it was found that the means are statistically different ($t(1272) = -6.81$, $p < .05$) which indicates that cleaning the trees does not only result in visual differences but also has a significant impact on the quantitative outputs.

3.3 Parameters Estimation

It was found that there is potential to use LiDAR-derived parameters for branch angle and internode length for the tropical tree model and branch angle for the Scots pine model. These parameters were extracted from the QSM outputs and were calculated separately for tree species and branch order (Fig. 5). The branch angle of the Scots pine had evenly distributed whiskers and a centred mean in the box plot. The tropical tree species are not always centred in the middle for the branch angles and internode lengths. The whiskers of the tropical trees are also longer on one size for the branching order and internode lengths of both branch orders, except for the first-order internode length Kabukalli. This can hint at skewed distributions for some tropical tree parameters. Additionally, the box plots show that there are outliers across all species, and more outliers are observed for the second-order parameters compared to the first-order.

3.4 Accuracy Analysis

The accuracy of the QSM outputs and estimated parameters were assessed using field observations and measurements from the manually corrected PCD, which we considered as ground truth. After the first inspection, it was found that for height the accuracy has an RMSE of 4.91 m and a relative RMSE of 26.40%. It was decided to see if the results

improved if the whole PCD was used when the maximum and minimum points were used. The RMSE improved to 2.45 m and a relative RMSE of 13.19%. This was also tested for the tropical trees, however, the accuracy did not improve so the QSM calculated height was used.

The tropical trees had higher RMSE for the 1st order branch angle (33.14 %) and DBH (17.24 %) parameters compared to the Scots pine trees, and lower RMSE (2.97 %) for the height (Table 4). Overall, the DBH for Scots pine trees had higher accuracy scores and the lowest accuracy was reported for the branch angle of the tropical trees. RMSE(%) of parameters for the second-order branches compared to the first-order branches were higher for the internode length of the tropical trees and branch angle of the Scots pine. The opposite was true for the branch angle of the first-order tropical tree branch angles. The QSM of the trees were inspected and examples of causes of errors were found, such as missing branches, misfit cylinders, and low quality TLS scans **[Supplementary Information Fig. S4]**.

The field observations and TLS-derived parameters were plotted against each other for visualisation and shown in Fig. 6. The plots in Fig. 6a show the results of the tropical trees. The height values are spread out and show discrepancies. The DBH is plotted around the 1:1 line. The branch angle plots for both branch orders show a large spread around the 1:1 and across small and large degrees. The internode line shows most values well fitted around the 1:1 line, with some large underestimation errors. The internode length for second-order branches shows more spread and show larger errors. The same spread is found back for the height plot as the tropical trees (Fig. 6b). Underestimations of TLS-derived height are observed for higher height values. The DBH values are plotted around the 1:1 line and the branch angle shows a close fit for both branch orders.

3.5 FSP Models with TLS-Derived Parameters

TLS-derived branch angle and internode length were used as inputs for the selected FSP models. The median, 25th-75th percentile and normal distribution function were given in separate pass files and the outputs were saved for each time step. The original input specified values of 0.3 to 0.7 cm, while ranges from the TLS-derived parameters varied between 36-231 cm. Putting the TLS-derived values in the model resulted in no growth of the trees. For the next runs, it was thus decided to only use the TLS-derived branch angle for the tropical tree model. The final input values for each tree species are specified in Table 5.

3.5.1 Tropical Tree Model

The tropical tree model was run for each species with the TLS-derived parameters. However, minor differences and the same trends were observed for the different variable outputs **[Supplementary Information Fig. S5]**. Because of the similarities, it was chosen to discuss the averaged outputs.

The variations in TLS-derived input parameters resulted in varying variable outputs compared to the original values (Fig. 7). The diameter variable output had slightly more conservative values for the TLS-derived branch angles compared to the default input values (-1% and -9%). Lower variable outputs were also observed for woody biomass (-1% and -17%) and crown area (-9% and -27%). Some TLS-derived input parameters also resulted in higher values compared to default models. For example, the TLS-derived minimum and normal distribution resulted in higher leaf biomass, with 25% and 12% respectively. The height started with differences in model inputs, but after around 100 years the height limit was reached and the variable outputs ended up the same. Overall, the minimum and normal distribution output variables differed the most compared to the default model outputs.

An additional three runs were performed with the Greenheart normal distribution parameters. The different runs resulted in the same trends for all variables, but the difference between output variables was not the same **[Supplementary Information Fig. S6]**. Diameter and height did not show large variations among the runs with the final diameter values having a standard deviation of 1.5 cm. The other parameters had larger variations of the final output parameter. The woody biomass showed a standard deviation of 457 mg and the leaf biomass 6093 g. The crown depth and area showed larger standard variations of 3.21 m and 38.34 m2 respectively.

The visual outputs of the tropical tree FSP model showed a similar growing pattern but differences in appearance (Fig. 8). The tree first grows up, with branches growing evenly along the stem. Once the maximum height is reached the branches closest to the ground will start to die until a distinguishable crown is formed. The TLS-derived parameter models show distinct branching patterns, except for the maximum branch angle. The tree with the median branch angle model inputs have branches that are more upward compared to the original values. The top of the crown is denser at 100, 150 and 200 years. The minimum branch angle tree time series show an even more upright branch angle compared to the median and the tree is slimmer as a result. Finally, the normal distribution tree output shows more random branches, and more distinguishable branches growing out of the tree. The tree is more asymmetric as a result.

3.5.2 Scots Pine Model

The TLS-derived inputs resulted in the same oscillating trend in all output variables, but differences in the output variable were found (Fig. 9). In contrast with the Tropical tree model, it was found that the output variables of the Scots pine model with TLS-derived inputs were observed to be higher compared to the default values. Only for TLS-derived height was

the default model variables found to be higher (-4% and -8%). The largest difference was found for root biomass where the TLS-derived normal distribution resulted in twice as much biomass compared to the default. The minimum and median TLS-derived parameters resulted in the largest differences overall.

Looking at the visual outputs of the FSP models there are also differences between the default and TLS-derived inputs models (Fig. 10). The default trees resulted in a sparser number of branches compared to the other models. The median and maximum TLS-derived input models do not show the gentle bending curve, which is present in the original, minGuo et al., 2012 and normal distribution models. The branches grow first horizontally before growing at a straight angle, resulting in a visually less realistic tree.

4 Discussion

Structural measurements of tree architecture are laborious to acquire with manual methods. Tree FSP models have high data needs and restraints in data acquisition limit model development. This research explored the potential of LiDAR data to serve as a reliable data source for acquiring non-destructive tree architecture measurements. Using LiDAR for FSP model development can provide parameters that are hard to obtain or previously not thought possible. To the author's knowledge, an overview of structural parameters used for FSP modellers has not been compiled before. The results of this research have the potential to serve as a guide for LiDAR data possibilities for FSP modellers and as a starting point for new areas of research for LiDAR. Additionally, no research has focused on using LiDAR-derived parameters for FSP model inputs and assessed the influence on the variable outcomes. Findings from this exploratory research highlight important considerations for the future, to ensure the successful integration of LiDAR data with FSP models.

4.1 FSP Model Parameter Needs and LiDAR Possibilities

Results from the literature review demonstrated the wide range of structural measurements used for FSP model development and the considerable extent of possibilities of LiDAR data to derive these. The large variety and varying scale of detail of the parameters highlight again the high data needs for FSP model development (Louarn and Song, 2020). LiDAR was found to be a possible reliable alternative for the parameters on the tree scale. LiDAR-derived DBH, height and woody biomass are already considered to be feasible to replace manual measurements for forest inventories (Aijazi et al., 2017; Jin et al., 2021). Bongers (2020) mentioned LiDAR data to be useful for detailed branch structures, which was also supported by the results of this study. However, it also became evident that at a certain level of detail, LiDAR becomes less reliable. Higher uncertainty is reported for parameters regarding smaller branches (Lau et al., 2018). In addition, parameters for internode and foliage geometry were not researched in large quantities. The reason could be because it was not deemed feasible, or these parameters are less relevant for current purposes (e.g. estimating biomass or wood quality). This highlights that there is still unexplored potential. Further research could decrease uncertainty for smaller tree structures and might bring forward new parameters which are feasible to be derived from LiDAR data by improving the trade-off among field data requirements, different LiDAR sensors (MLS, TLS, UAV), and LiDAR data collection strategy. For example, scanning fine structures might be resolved by using a denser scanning strategy, but requires more fieldwork time, processing power, even a high density LiDAR scanner.

An essential question regarding the accuracy is what error rate is acceptable for FSP model development, this is also highlighted by Calder, et al. (2020). The review did not have an accuracy standard which the results of the papers had to meet for it to be included. This

resulted in parameters being marked as possible but the large rates of errors might be unsatisfactory. For example, the whorl detection was 70% accurate, which for some fields of research is considered a high error rate. There is yet no clear standard to which the accuracy needs to be upheld and will need further research to define acceptable error rates. Furthermore, a tick mark does not mean that the accuracies are repeatable for different tree species and scanning circumstances. For example, the internode length of cotton plants was researched by Saeed and Li (2021). When the same method is applied to larger trees the occlusion or distance from the scanner could lower the accuracies. Additionally, Pallas et al. (2020) scanned 2 and 3-year-old apple trees in an orchard to estimate shoot lengths. In a dense forest, such detail might not be possible to capture.

The average number of structural measurements used for the development of FSP models was found to be 11 parameters. When analysing the LiDAR literature it was found that most methods specialise in retrieving only one or a handful of parameters. When a larger number of parameters are to be derived, different methods and programs are required (Georgi et al., 2018), which can be time-consuming and would require expert knowledge. For the review, the QSM by Raumonen et al. (2013) was included to also highlight the possibility of one general method to acquire multiple parameters at once. One downside of the QSM is that foliage is not included and thus no information can be retrieved from this. Other tree modelling methods could also be examined to explore if they could retrieve more parameters.

An advantage of LiDAR is that measurements can be done non-destructively. However, from the review, it came forward that tree age is important information that is needed to accompany the other parameter measurements. Knowledge will need to be available of the stand age or an estimate could be made based on the tree characteristics. Forest stand age estimation was performed by Rizeei et al. (2018), but this was for one species and needed calibration. Also, tree species are crucial information that needs to

accompany the LiDAR measurements. Åkerblom et al. (2017) made good estimates of multiple tree species and also recent advancements using deep learning show promising results (Allen et al., 2023), yet species recognition models will need additional training data when other species are to be included.

The scope of this review was on FSP models about non-fruit bearing trees. Nevertheless, fruit bearing FSP models have a large share in the amount of tree FSP models (O'Sullivan et al., 2021). This was confirmed in our systematic literature search, where the non-fruit bearing criteria excluded more than one-third of the screened papers. This means that there could be parameters missing from the overview which are essential for a large share of tree FSP models. The aim of fruit-bearing tree models is often different from non-fruit bearing trees, which results in different structural parameter needs. FSP models for fruit-bearing trees are made to make management decisions for improved crop growth, which is a process not included in the review. For instance, the model developed by Boudon et al. (2020) describes the geometry of the fruits and uses the number of fruits produced for validation. For these specific types of models, an additional review might be necessary. For the scope, it was also decided to only include papers that had information regarding descriptions of structural measurements done for the study. As a result, a substantial amount of papers were excluded as they used databases of previously done measurements. By doing so, parameters that tend to not be measured often are not included. Even though they might be the parameters which are the ones being most difficult to acquire and thus benefit most to have an alternative measuring method. For new studies, updating the literature review to include new papers would help to fill this gap.

In conclusion, the literature review demonstrated good potential for LiDAR data to support FSP model development. However, it is important to consider the limitations of accuracy for smaller-scale parameters. The main focus of LiDAR-derived parameters have

been on forest inventories, so information regarding foliage and branch structures are less researched. Additionally, a standard for the accuracy requirements for structural parameters should be defined to determine if the error rates of LiDAR-derived measurements are acceptable. LiDAR research for extracting parameters is also still relatively young and will likely make big leaps in the coming years. New advancements are being made with LiDAR scanners using multispectral scans or combining LiDAR remote sensing data (Van Leeuwen and Nieuwenhuis, 2010). Also, the use of machine learning or deep learning shows a large potential for retrieving more useful data (Saeed and Li, 2021).

4.2 Accuracy of TLS-Derived Parameters

Model quality was assessed by estimating height and DBH. The accuracy of the derived parameters varied between the tropical tree and the Scots pine data sets. Tree height RMSE for tropical trees (3.4 m) and Scots pine (2.45 m) were both worse compared to Y. Wang et al. (2019) (1.68 - 2.11 m) and Liu et al. (2018) (0.54 - 1.23 m). Height errors were distributed along all size classes for the tropical trees, whereas errors in the Scots pine parameters were caused by underestimation for larger trees. This problem was also found by Y. Wang et al. (2019) to be a large source of error. The errors are likely caused by the laser not being able to reach the top of the crown because of occlusion. Although, lower accuracies can also be caused by errors in the field measurements (Luoma et al., 2017). Vaccari et al. (2013) mentioned the tool used for field observations for acquiring the Scots pine height introduced bias, which could be a source of error. DBH for tropical trees (RMSE: 12.96 cm) showed substantially higher errors compared to the Scots pine (RMSE: 1.17 cm). High errors for DBH of tropical trees were also reported by Lau et al. (2019a) who found that buttresses were a source of error. The derived DBH from the Scots pine LiDAR data was more accurate

compared to Brede et al. (2017) (RMSE: 4.24 cm) and Pyörälä et al. (2018) (RMSE: 1.31 cm).

The accuracy of the branch angle estimation for Scots pine (RMSE(%): 12.97%-19.11%) was higher compared to tropical tree branch angles (RMSE(%): 46.11%-34.32%). Scots Pine branch angle accuracy was also higher compared to the methods of Côté et al. (2011) (RMSE(%): 25%) and Pyörälä et al. (2018) (RMSE(%): 23.4%). However, for their structural measurements, they took all branches attached to the stem, instead of the first branch of each tree. Branches closer to the ground have fewer problems of occlusion and are generally larger which makes them more accurate in the QSM PCD (Malhi et al., 2018). This could explain the higher accuracy reported for this study, as more errors are reported when also measuring branches higher in the crown. In the future, it is thus relevant to assess the accuracy by taking multiple branches from a tree at different heights. The high errors for the tropical trees' branch angle were likely caused by taking only the first cylinder of a branch and one cylinder from the trunk. Individual cylinders often fit in slightly different directions to account for curvatures in the branch which makes the branch angle sensitive if only one cylinder is considered. A solution could be to take the average of multiple cylinders (Malhi et al., 2018).

Mistakes in the QSM led to large errors for internode length (RMSE: 118.62 cm). Saeed and Li (2021) reported an RMSE of 1.04 cm which is substantially better than the found RMSE of this research. Even though the plants studied were largely different (max. tree height of 1.5 m against 44.2 m) the errors of this research are still substantial. For internode length, all large errors were caused by extra, or missing cylinders of the branches. This indicates that the QSM approach might not be the best method for estimating internode length, and a deep learning approach (Saeed and Li, 2021) could give better estimates. Internode allometric models are found to not be constant for the whole tree, so further

research in this area is important to help create more accurate estimates for FSP models (Diao et al., 2014).

Effect of manually correcting the PCD before the QSM shows minimal changes for tree parameters that are often used for forest inventories. Yet, for higher detailed branch structure parameters it was found to have a significant effect on QSM outputs. If LiDAR will be used in the future it is thus good to consider that preprocessing steps have large effects on the outputs. Visually it was found to reduce noise and make branches more distinguished, but additional research is needed to assess if accuracy also improves. Since manually correcting the data is a laborious process (45 minutes per tree) it is also essential to assess the payoff between scalability and data quality. During manually correcting there is also a loss of detail as smaller branches are often surrounded by foliage. This ties in again with the point made in section 4.1, where more research is needed to determine the sensitivity of errors of the LiDAR-derived parameters for FSP model performance. Additionally, segmentation of the trees from a PCD has a large effect on QSM results. For this study we used pre-segmented trees which were also manually corrected. Fully automatic segmentation algorithms exist with promising results (Krisanski et al., 2021, Wilkes et al., 2023), but errors still occur. Thus the trade-off of fully automizing and manually correcting at different steps of the preprocessing also need to be further explored.

The quality of the QSM is also highly dependent on the PCD density and scanning conditions (occlusion). The data used for this research came from a densely populated tropical forest and was scanned in conditions that did not result in optimal PCD density (Lau et al., 2018). Additionally, the scanned trees in this study had foliage which led to the occlusion of the smaller branches. Deciduous trees in winter or dead trees which has shed their leaves could be scanned in the future to mitigate this. More research is needed for designing a pipeline that gets the most accurate results but is also fast and does not require

laborious manual measurements. Fully automatic pipelines are still found to be unreliable and intermediate checks are crucial to avoid large errors (Martin-Ducup et al., 2021). For this research, only first and second-order branches were considered, but higher-order branches could also be relevant for FSP models. QSM does not perform well in identifying and counting third and fourth-order branches (C. Zhang et al., 2020), so further research would be needed to assess the accuracy.

The advantage of using a QSM is that it is scalable since it performs well with larger samples of trees (Raumonen et al., 2015). However, the accuracy of the QSM is dependent on the quality of the LiDAR input data and the accuracies found for this research did not always compare to skeletonization methods. For future research, accuracy could be improved by exploring other 3D tree reconstruction methods which could overcome certain limitations of LiDAR data. Different reconstruction methods are found to perform better under different scan circumstances or parameters to be estimated (Bournez et al., 2017). For example, the skeletonization method by Côté et al. (2011) could be a good alternative modelling approach, as the effect of occlusion is accounted for and information regarding the geometry of foliage is included. These different reconstruction methods could be assessed to find better optimal performing methods (Boudon et al., 2014). Additionally, measurements of branch angle and internode length were only done for one branch per tree. A more complete study would be needed to make conclusions about the accuracy of internode length and branch angle for the whole tree, and what method would result in the highest accuracy.

4.3 LiDAR-derived Inputs in FSP Models

Branch angle was successfully used as inputs in both FSP models, but internode length resulted in problematic outcomes. This result is in contrast with the literature (Section 3.1), where internode length was reported to be derivable from LiDAR data and could potentially

replace manual measurements. The internode length was considerably larger than the default value used in the model, which could be the reason why the model did not work. The difference between the default and the LiDAR-derived value could be explained by the fact that the internode lengths during the first years of the tree growth are different from the mature trees measured for this study. Thus it might not be possible to use LiDAR-derived internode length as model inputs, even though this was found to be feasible in the literature review. The definition of internode distance could also be different depending on the models. The model of Petter et al. (2021) defined nodes as points on the stem where branches grow from. However, nodes can also be defined differently across research fields. Botanical nodes are defined as the point of the stem where buds or leaves originate from. Further research will need to explore if such detailed tree organs can also be detected from a LiDAR scan.

LiDAR-derived branch angle inputs were different from the default inputs and the tropical tree model runs with LiDAR-derived branch angle resulted in lower variable outputs compared to the default values. This contrasts the Scots pine model, for which the LiDAR-derived branch angle resulted in higher variable outputs. Perez et al. (2018) found that TLS could provide good validation data, but also highlights that discrepancies in the TLS validation data make it difficult to distinguish errors in the model and the validation data. This highlights the need for caution when using TLS data since errors in the data can have a substantial effect on model outputs. However, TLS data is much more scalable than manual measurements, allowing for larger sample sizes. More research is needed to conclude if the results of output variables are close to observations in reality.

Differences were found in variable output between TLS-derived inputs and default values, indicating model sensitivity to changes in branch angle. Streit et al. (2016) also looked at the sensitivity of the LIGNUM Scots pine model with differing branch angles. It was found that first-order branch angles had limited effect related to light interception, and

higher-order branch angles had minimal effect. The range of branch angles for the sensitivity analysis was 4.5 degrees. This choice was not based on data but on an assumption that 10% ranges from the set branch angle were reasonable. Results from the QSM suggest that there might be larger ranges, and including normal distribution might lead to larger changes than observed before. In this research, both branch orders were changed during the model running so it could be relevant to look at the sensitivity of them separately in the future.

Visually the normal distribution of TLS-derived model inputs reflects the non-asymmetric branch architecture well. Probability distribution functions are more often used for branch angles to reflect the stochasticity during the growth of the tree (Lu et al., 2011; Parsons et al., 2011; Potapov et al., 2017). Using LiDAR can provide large quantities of measured attributes, from which distribution can be made to make more realistic models. This finding could be interesting to consider for the inclusion of LiDAR-derived parameter distributions for general tree models, which can be reparametrized more easily for different species (Henke et al., 2016).

The visual output of the branching structure changed for different TLS-derived inputs in the Scots pine model. The median and maximum values created sharp angles at the stem and straight branches. An explanation could be that occlusion and distance from the LiDAR scanner resulted in fewer branch angles measured at the top of the crown and the end of branches. The LIGNUM model requires the branch angle for new shoots, which are thus the places where fewer measurements were possible. After each time step, there is a bending effect that brings the branches down to a maximum of 90 degrees. The branch angles measured from the Scots pines were already branches that had bent which could be the reason that the bending effect was not used with larger values of TLS-derived branch angles. Acquiring a branching angle for new shoots in the tree crown with foliage might not be suitable with LiDAR because of occlusion and higher uncertainty for smaller branches. In the

future, it could be interesting to use UAV-based laser scanning data, which can measure the top of the crown directly and reduce the influence of occlusion. An alternative approach is to find trees that have lost the needles or leaves to acquire better detail in the crown (Aijazi et al., 2017).

Results from this research confirm the statement made by Beyer et al. (2017), who claimed that LiDAR can be of great value for FSP model development. However, LiDAR-derived model inputs differed from the default values, resulting in different output variables. Additionally, not all input values are feasible with LiDAR, as it was found that internode length derived from LiDAR could not be used as FSP model input. More research is needed in the future to understand the effect of using alternative derived inputs for FSP models. Results also showed that using LiDAR can result in a scalable and efficient method for deriving a large range of parameters for a large tree sample or individual tree. This can be useful for a general tree model that requires species-specific inputs for a large range of species or to personalize specie specific FSP models with data under different environmental conditions. FSP models are also becoming more accessible, with new tools like Jupyter notebooks (Vaillant et al., 2022), which will also likely increase the demand for tree architectural data. For this research focus was put on FSP model inputs that could be derived from a single time measurement, e.g. parameters that do not change over time. Nonetheless, models also need inputs that are related to the dynamic growth rules of the tree. Because of the non-destructive measurements taken by LiDAR, it could also be possible to track change through a time series (Liang et al., 2012). Although, LiDAR time series for multiple years exists (Campos et al., 2021, Calders et al., 2023), logistical challenges arise and might not be feasible in most time frames. Sievänen et al. (2018) overcame this by creating a pseudo-time series by scanning trees of different ages.

5 Conclusion

Tree FSP models have high data needs because of the inclusion of both the 3D architecture and functional processes. As a result, acquiring structural measurements of trees with conventional methods is a laborious process. LiDAR has been mentioned to be a possible reliable measurement tool that can be used for FSP model development. There has been some research where LiDAR for validation and parametrization, but these were for specific cases. The aim of this research was to create an overview of the possibilities of LiDAR data to complement tree FSP model development and to investigate if TLS-derived tree traits could be used for tree FSP model inputs.

It was demonstrated through a literature review that LiDAR could be used as an alternative measurement tool for a large share of parameters used in FSP models. Important considerations were found that need to be addressed before LiDAR can be used as a reliable data source. There is still large uncertainty surrounding the accuracy of smaller scale parameters, like internode and foliage details. Additionally, accuracy standards need to be defined for FSP model data sources, to make sure that data quality is satisfactory.

Two parameters, branch angle and internode length were found to have potential as TLS-derived model inputs. However, the accuracy of the TLS-derived parameters was variable because of errors in the QSM fitting. TLS-derived branch angles were successfully used as input parameters in both FSP models. On the other hand, TLS-derived internode lengths were not found suitable which contradicted the results from the literature review. Using the TLS-derived inputs resulted in different output variables of the FSP models compared to the default models. Visually there were also differences, and variations of TLS-derived inputs resulted in different architectural outcomes. It was concluded that it is possible to use TLS-derived branch angle as FSP model input, but further research is necessary to understand the implications on model outcomes.

The results demonstrated that there is considerable potential for LiDAR data to complement FSP models but some considerations still need to be further worked out before LiDAR can be used as a reliable alternative data source.

1.) What accuracy standard do LiDAR-derived parameters need to attain to be acceptable for FSP model development?

2.) Which methods work best for deriving different types of parameters, and how can non-LiDAR experts use these?

3.) What effect does erroneous data have on FSP model outcomes?

Conclusions from this research have resulted in new insights into considerations and limitations of using LiDAR for deriving structural parameters and can further advance finding new possibilities for interdisciplinary research between the research fields of LiDAR and FSP modelling. In the future, LiDAR could help improve efficiency in building new FSP models, increase the accuracy of existing models, add metrics for optimization, and open up new possibilities to explore previously unobtainable plant traits to include in the models.

Data availability statement

The data underlying this article are available in 4TU.Centre for Research Data, at https://dx.doi.org/10.4121/2b7e832f-12e9-4d0e-92e2-5aef9bcf9142.

Supporting Information

Figure S1 Schematic overview of the methodology with associated sections

Figure S2 Classification results of a tropical tree (Parkia pendula) 100_16 using different feature similarity parameters (Nzthres). The woody component is classified in red and the

soft component is classified in blue. The tree used for data exploration and not included in the final analysis.

Figure S3 Analysis results for choosing the optimal feature similarity parameter (N_{zthres}) used in the LeWoS wood-leaf separation method. The overall score is the score for wood detail minus the number of leaves included.

Figure S4 Selection of examples of errors encountered when inspecting the QSM results. a.) The branch was not included in the QSM, resulting in an internode length error; b.) and c.) Multiple cylinders fitted wrong, which affects the branch angle and internode length; and d.) Low-quality LiDAR scan resulting in large overall errors.

Figure S5 Tropical tree FSP model selected parameter output for different LiDAR-derived parameter inputs. Minimum-Maximum/Median, normal distribution for first and second order branches.

Figure S6 Outputs of three runs with the same LiDAR-derived normal distribution function of a Greenheart tree (first-order : 60 − 10/42, μ = 32, σ = 38) (second-order : 39 − 81/60, μ = 64, σ= 35).

Table S1: Selection of FSP models developed in GroIMP that were considered for the analysis. NA= not available.

Table S2: Scoring points and descriptions that were used for assessing and choosing the optimal Nzthres score in the LeWoS algorithm of D. Wang et al. (2020).

Table S3: Structural measurements are mentioned in the methodology of a selection of tree FSP models. Papers were selected based on a systematic literature search.

Table S4: Variable output results with different LiDAR-derived branch angle inputs using the Tropical tree FSP model from Petter et al. (2021). The default model inputs are compared to the 4 different LiDAR-derived branch angles by calculating the change.

Table S5 Variable output results with different LiDAR-derived branch angle inputs using the Scots pine FSP model from Smolevnova et al. (2013). The default model inputs are compared to the 4 different LiDAR-derived branch angles by calculating the change.

Funding information
Our Guyana laser scanning and field data were acquired through collaboration between Wageningen University and Guyana Forestry Commission, supported by the donors to the CGIAR Fund, SilvaCarbon research project 14-IG-11132762-350 and ERA-GAS NWO-3DforMod project 5160957540

Acknowledgements

We give special thanks to Katarína Streit who provided us with the code for the Scots Pine FSP model. Additionally, we give thanks to Harm Bartholomeus for measuring and laser scanning the trees in Loobos.

References

Aijazi AK, Checchin P, Malaterre L, Trassoudaine L. 2017. Automatic detection and parameter estimation of trees for forest inventory applications using 3D terrestrial LiDAR. *Remote Sensing* 9 (9): 946.

Åkerblom M, Raumonen P, Mäkipää R, Kaasalainen M. 2017. Automatic tree species recognition with quantitative structure models. *Remote Sensing of Environment* 191: 1–12.

Allen MJ, Grieve SW, Owen HJ, Lines ER. 2023. Tree species classification from complex laser scanning data in Mediterranean forests using deep learning. *Methods in Ecology and Evolution* 14 (7): 1657-1667.

Bailey BN. 2019. Helios: A scalable 3D plant and environmental biophysical modeling framework. *Frontiers in Plant Science* 10: 1185.

Baltsavias EP. 1999. Airborne laser scanning: Basic relations and formulas. *ISPRS Journal of Photogrammetry and Remote Sensing* 54 (2-3): 199–214.

Barbault N, Dupraz C, Lauri PE, Gosme M. 2024. Insights into fruit tree models relevant to simulate fruit tree-based agroforestry systems. *Agroforestry Systems* 98 (4): 817-835.

Beyer R, Bayer D, Letort V, Pretzsch H, Cournède PH. 2017. Validation of a functional-structural tree model using terrestrial Lidar data. *Ecological Modelling* 357: 55–57.

Blackman VH. 1919. The compound interest law and plant growth. *Annals of Botany* 33 (131): 353–360.

Bongers FJ. 2020. Functional-structural plant models to boost understanding of complemen tarity in light capture and use in mixed-species forests. *Basic and Applied Ecology* 48: 92–101.

Boudon F, Preuksakarn, C, Ferraro, P, et al. 2014. Quantitative assessment of automatic reconstructions of branching systems obtained from laser scanning. *Annals of Botany* 114 (4): 853–862.

Boudon F, Persello S, Jestin A, et al. 2020. V-mango: A functional–structural model of mango tree growth, development and fruit production. *Annals of Botany* 126 (4): 745–763.

Bournez E, Landes T, Saudreau M, Kastendeuch P, Najjar G. 2017. From TLS point clouds to 3D models of trees: a comparison of existing algorithms for 3D tree reconstruction. *3D Virtual Reconstruction and Visualization of Complex Architectures* 42 (2): 113–120.

Brede B, Lau A, Bartholomeus HM, Kooistra L. 2017. Comparing RIEGL RiCOPTER UAV LiDAR Derived Canopy Height and DBH with Terrestrial LiDAR. *Sensors* 17 (10): 2371.

Bucksch A, Fleck S. 2011. Automated detection of branch dimensions in woody skeletons of fruit tree canopies. *Photogrammetric Engineering & Remote Sensing* 77 (3): 229–240.

Buck-Sorlin G, Kniemeyer O, Kurth W. 2008. A Model of Poplar (Populus sp.) Physiology and Morphology Based on Relational Growth Grammars. *Mathematical Modeling of Biological Systems, Volume II*: 313–322.

Calders K, Newnham G, Burt A, et al. 2015a. Nondestructive estimates of above-ground biomass using terrestrial laser scanning. *Methods in Ecology and Evolution* 6 (2): 198–208.

Calders K, Schenkels T, Bartholomeus H, Armston J, Verbesselt J, Herold M. 2015b. Monitoring spring phenology with high temporal resolution terrestrial LiDAR measurements. *Agricultural and Forest Meteorology* 203: 158–168.

Calders K, Origo N, Burt A, et al. 2018. Realistic forest stand reconstruction from terrestrial LiDAR for radiative transfer modelling. *Remote Sensing* 10 (6): 933.

Calders K, Adams J, Armston J, et al. 2020. Terrestrial laser scanning in forest ecology: Expanding the horizon. *Remote Sensing of Environment* 251: 112102.

Calders K, Brede B, Newnham G, et al. 2023. StrucNet: a global network for automated vegetation structure monitoring. *Remote Sensing in Ecology and Conservation* 9 (5): 587-598.

Combes D, Chelle M, Sinoquet H, Varlet-Grancher C. 2008. Evaluation of a turbid medium model to simulate light interception by walnut trees (hybrid NG38× RA and Juglans regia) and sorghum canopies (Sorghum bicolor) at three spatial scales. *Functional Plant Biology* 35 (10): 823-836.

Campos MB, Litkey P, Wang Y, et al. 2021. A long-term terrestrial laser scanning measurement station to continuously monitor structural and phenological dynamics of boreal forest canopy. *Frontiers in Plant Science* 11: 606752.

Côté JF, Widlowski JL, Fournier RA, Verstraete MM. 2009. The structural and radiative consistency of three-dimensional tree reconstructions from terrestrial lidar. *Remote Sensing of Environment* 113 (5): 1067–1081.

Côté JF, Fournier RA, Egli R. 2011. An architectural model of trees to estimate forest structural attributes using terrestrial LiDAR. *Environmental Modelling & Software* 26 (6): 761–777.

Crimaldi M, Cartenì F, Giannino F. 2021. Vismaf: synthetic tree for immersive virtual visualization in smart farming. Part i: scientific background review and model proposal. *Agronomy* 11 (12): 2458.

DeJong TM, Da Silva D, Vos J, Escobar-Gutiérrez AJ. 2011. Using functional–structural plant models to study, understand and integrate plant development and ecophysiology. *Annals of Botany* 108 (6): 987–989.

Delagrange S, Rochon P. 2011. Reconstruction and analysis of a deciduous sapling using digital photographs or terrestrial-LiDAR technology. *Annals of Botany* 108 (6): 991–1000.

Delagrange S, Jauvin C, Rochon P. 2014. PypeTree: A tool for reconstructing tree perennial tissues from point clouds. *Sensors* 14 (3): 4271–4289.

Diao J, Lei X, Wang J, et al. 2014. Quantifying the Variability of Internode Allometry within and between Trees for Pinus tabulaeformis Carr. Using a Multilevel Nonlinear Mixed-Effect Model. *Forests* 5 (11): 2825–2845.

Disney M, Lewis P, Saich P. 2006. 3D modelling of forest canopy structure for remote sensing simulations in the optical and microwave domains. *Remote Sensing of Environment* 100 (1): 114–132.

Disney M. 2019. Terrestrial lidar: A three-dimensional revolution in how we look at trees. *New Phytologist* 222 (4): 1736–1741.

Dorji Y, Schuldt B, Neudam L, et al. 2021. Three-dimensional quantification of tree architecture from mobile laser scanning and geometry analysis. *Trees* 35: 1385–1398.

Dornbusch T, Wernecke P, Diepenbrock W. 2007. A method to extract morphological traits of plant organs from 3D point clouds as a database for an architectural plant model. *Ecological Modelling* 200 (1-2): 119–129.

Evers JB, Van Der Werf W, Stomph TJ, Bastiaans L, Anten NP. 2019. Understanding and optimizing species mixtures using functional–structural plant modelling. *Journal of Experimental Botany* 70 (9): 2381–2388.

Fernández-Sarría A, López-Cortés I, Estornell J, Velázquez-Martí B, Salazar D. 2019. Estimating residual biomass of olive tree crops using terrestrial laser scanning. *International Journal of Applied Earth Observation and Geoinformation* 75: 163–170.

Gaudio N, Escobar-Gutiérrez AJ, Casadebaig P, et al. 2019. Current knowledge and future research opportunities for modeling annual crop mixtures. a review. *Agronomy for Sustainable Development* 39 (2): 1–20.

Georgi L, Kunz M, Fichtner A, et al. 2018. Long-term abandonment of forest management has a strong impact on tree morphology and wood volume allocation pattern of European beech (Fagus sylvatica L.). *Forests* 9 (11): 704.

Grisafi F, DeJong TM, Tombesi S. 2022. Fruit tree crop models: an update. *Tree Physiology* 42 (3): 441-457.

Guo H, Lei X, Cournede PH, Letort V. 2012. Characterization of the effects of inter-tree competition on source–sink balance in Chinese pine trees with the GreenLab model *Trees* 26 (3): 1057–1067.

Hanan J. 1997. Virtual plants—integrating architectural and physiological models. *Environmental Modelling & Software* 12 (1): 35–42.

Hemmerling R, Kniemeyer O, Lanwert D, Kurth W, Buck-Sorlin G. 2008. The rule-based language XL and the modelling environment GroIMP illustrated with simulated tree competition. *Functional Plant Biology* 35 (10): 739–750.

Henke M, Kurth W, Buck-Sorlin GH. 2016. FSPM-P: Towards a general functional structural plant model for robust and comprehensive model development. *Frontiers of Computer Science* 10 (6): 1103–1117.

Jackson T, Shenkin A, Kalyan B, et al. 2019. A new architectural perspective on wind damage in a natural forest. *Frontiers in Forests and Global Change* 1: 13.

Jin S, Sun X, Wu F, et al. 2021. Lidar sheds new light on plant phenomics for plant breeding and management: Recent advances and future prospects. *ISPRS Journal of Photogrammetry and Remote Sensing* 171: 202–223.

Kang M, Hua J, Wang X, De Reffye P, Jaeger M, Akaffou S. 2018. Estimating sink parameters of stochastic functional-structural plant models using organic series-continuous and rhythmic development. *Frontiers in Plant Science* 9: 1688.

Klemmt HJ, Seifert T, Seifert S, Kunneke A, Wessels B, Pretzsch H. 2010. Assessment of branchiness in a Pinus pinaster plantation by terrestrial laser scanner data as a link between exterior and interior wood properties. *Proceedings of SilviLaser 2010*: 253-264.

Kniemeyer O. 2008. *Design and implementation of a graph grammar based language for functional structural plant modelling*. PhD Thesis, Brandenburg University of Technology, Germany.

Krisanski S, Taskhiri MS, Gonzalez Aracil S, et al. 2021. Forest structural complexity tool—an open source, fully-automated tool for measuring forest point clouds. *Remote Sensing* 13 (22): 4677.

Lau A, Bentley LP, Martius C, et al. 2018. Quantifying branch architecture of tropical trees using terrestrial LiDAR and 3D modelling. *Trees* 32 (5): 1219–1231.

Lau A, Calders K, Bartholomeus H, et al. 2019a. Tree biomass equations from terrestrial LiDAR: a case study in Guyana. *Forests* 10 (6): 527.

Lau A, Martius C, Bartholomeus H, et al. 2019b. Estimating architecture-based metabolic scaling exponents of tropical trees using terrestrial LiDAR and 3D modelling. *Forest Ecology and Management* 439: 132–145.

Liang X, Hyyppä J, Kaartinen H, Holopainen M, Melkas T. 2012. Detecting changes in forest structure over time with bi-temporal terrestrial laser scanning data. *ISPRS International Journal of Geo-Information* 1 (3): 242–255.

Liang X, Kankare V, Hyyppä J, et al. 2016. Terrestrial laser scanning in forest inventories. *ISPRS Journal of Photogrammetry and Remote Sensing* 115: 63–77.

Liu G, Wang J, Dong P, Chen Y, Liu Z. (2018). Estimating individual tree height and diameter at breast height (DBH) from terrestrial laser scanning (TLS) data at plot level. *Forests* 9 (7): 398.

Louarn G, Song Y. 2020. Two decades of functional–structural plant modelling: now addressing fundamental questions in systems biology and predictive ecology. *Annals of Botany* 126 (4): 501–509.

Lu M, Nygren P, Perttunen J, Pallardy SG, Larsen DR. 2011. Application of the functional-structural tree model LIGNUM to growth simulation of short-rotation eastern cottonwood. *Silva Fennica* 45: 431–474.

Luoma V, Saarinen N, Wulder MA, et al. 2017. Assessing precision in conventional field measurements of individual tree attributes. *Forests* 8 (2): 38.

Ma L, Zheng G, Eitel JU, Magney TS, Moskal LM. 2017. Retrieving forest canopy extinction coefficient from terrestrial and airborne lidar. *Agricultural and Forest Meteorology* 236: 1–21.

Malhi Y, Jackson T, Patrick Bentley L, et al. (2018). New perspectives on the ecology of tree structure and tree communities through terrestrial laser scanning. *Interface Focus* 8 (2), 20170052.

Mallet C, Bretar F. 2009. Full-waveform topographic lidar: State-of-the-art. *ISPRS Journal of Photogrammetry and Remote Sensing* 64 (1): 1–16.

Martin-Ducup O, Mofack G, Wang D, et al. (2021). Evaluation of automated pipelines for tree and plot metric estimation from TLS data in tropical forest areas. *Annals of Botany* 128 (6): 753– 766.

Moher D, Liberati A, Tetzlaff J, Altman DG, PRISMA Group. 2009. Preferred reporting items for systematic reviews and meta-analyses: the PRISMA statement. *PLoS medicine* 6 (7): e1000097.

Newnham GJ, Armston JD, Calders K, et al. 2015. Terrestrial laser scanning for plot-scale forest measurement. *Current Forestry Reports* 1 (4) 239–251.

O'Sullivan H, Raumonen P, Kaitaniemi P, Perttunen J, Sievänen R. 2021. Integrating terrestrial laser scanning with functional–structural plant models to investigate ecological and evolutionary processes of forest communities. *Annals of Botany* 128 (6): 663-684.

Pallas B, Martinez S, Simler O, Carrie E, Costes E, Boudon F. 2020. Assessing T-LiDAR technology for high throughput phenotyping apple tree topological and architectural traits. *Acta Horticulturae* 1281: 625–632.

Parsons RA, Mell WE, McCauley P. 2011. Linking 3D spatial models of fuels and fire: Effects of spatial heterogeneity on fire behavior. *Ecological Modelling* 222 (3): 679–691.

Paulus S, Schumann H, Kuhlmann H, Léon J. 2014. High-precision laser scanning system for capturing 3D plant architecture and analysing growth of cereal plants. *Biosystems Engineering* 121: 1–11.

Perez RP, Costes E, Théveny F, Griffon S, Caliman JP, Dauzat J. 2018. 3D plant model assessed by terrestrial LiDAR and hemispherical photographs: A useful tool for comparing light interception among oil palm progenies. *Agricultural and Forest Meteorology* 249: 250–263.

Perttunen J, Sievänen R, Nikinmaa E. 1998. LIGNUM: a model combining the structure and the functioning of trees. *Ecological modelling* 108 (1-3): 189–198.

Petter G, Kreft H, Ong Y, Zotz G, Cabral JS. 2021. Modelling the long-term dynamics of tropical forests: From leaf traits to whole-tree growth patterns. *Ecological Modelling* 460: 109735.

Popescu SC, Wynne RH, Nelson RF. 2003. Measuring individual tree crown diameter with lidar and assessing its influence on estimating forest volume and biomass. *Canadian Journal of Remote Sensing* 29 (5): 564–577.

Popescu SC, Zhao K. 2008. A voxel-based lidar method for estimating crown base height for deciduous and pine trees. *Remote Sensing of Environment* 112 (3): 767–781.

Potapov I, Järvenpää M, Åkerblom M, Raumonen P, Kaasalainen M. 2017. Bayes Forest: a data-intensive generator of morphological tree clones. *GigaScience* 6 (10): gix079.

Pyörälä J, Liang X, Vastaranta M, et al. 2018. Quantitative Assessment of Scots Pine (Pinus Sylvestris L.) Whorl Structure in a Forest Environment Using Terrestrial Laser Scanning. *IEEE Journal of Selected Topics in Applied Earth Observations and Remote Sensing* 11 (10): 3598–3607.

Raumonen P, Kaasalainen M, Åkerblom M, et al. 2013. Fast automatic precision tree models from terrestrial laser scanner data. *Remote Sensing* 5 (2): 491–520.

Raumonen P, Casella E, Calders K, Murphy S, Åkerblom M, Kaasalainen M. 2015. Massive-scale tree modelling from TLS data. *ISPRS Annals of the Photogrammetry, Remote Sensing and Spatial Information Sciences* 2 (3): 189-196.

Ritter T, Schwarz M, Tockner A, Leisch F, Nothdurft A. 2017. Automatic mapping of forest stands based on three-dimensional point clouds derived from terrestrial laser scanning. *Forests* 8 (8): 265.

Rizeei HM, Shafri HZ, Mohamoud MA, Pradhan B, Kalantar B. 2018. Oil palm counting and age estimation from WorldView-3 imagery and LiDAR data using an integrated OBIA height model and regression analysis. *Journal of Sensors* 2018: 1–13.

Saeed F, Li C. 2021. Plant organ segmentation from point clouds using Point-Voxel CNN. *2021 ASABE Annual International Virtual Meeting:* 1.

Sievänen R, Nikinmaa E, Nygren P, Ozier-Lafontaine H, Perttunen J, Hakula H. 2000. Components of functional-structural tree models. *Annals of forest science* 57 (5): 399–412.

Sievänen R, Perttunen J, Nikinmaa E, Kaitaniemi P. 2008. Toward extension of a single tree functional–structural model of Scots pine to stand level: effect of the canopy of randomly distributed, identical trees on development of tree structure. *Functional Plant Biology* 35 (10): 964–975.

Sievänen R, Godin C, DeJong TM, Nikinmaa E. 2014. Functional–structural plant models: A growing paradigm for plant studies. *Annals of Botany* 114 (4): 599–603.

Sievänen R, Raumonen P, Perttunen J, Nikinmaa E, Kaitaniemi P. 2018. A study of crown development mechanisms using a shoot-based tree model and segmented terrestrial laser scanning data. *Annals of Botany* 122 (3): 423–434.

Smoleňová K, Henke M, Ong Y, Kurth W. 2013. Rule-based integration of LIGNUM into GroIMP. *Proceedings of the 7th International Conference on Functional-Structural Plant Models*: 214–216.

Stovall AE, Vorster AG, Anderson RS, Evangelista PH, Shugart HH. 2017. Non-destructive aboveground biomass estimation of coniferous trees using terrestrial LiDAR. *Remote Sensing of Environment* 200: 31–42.

Stovall AE, Masters B, Fatoyinbo L, Yang X. 2021. TLSLeAF: automatic leaf angle estimates from single-scan terrestrial laser scanning. *New Phytologist* 232 (4): 1876-1892.

Streit K, Henke M, Bayol B, Cournède PH, Sievänen R, Kurth W. 2016. Impact of geometrical traits on light interception in conifers: analysis using an FSPM for Scots pine. *2016 IEEE International Conference on Functional-Structural Plant Growth Modeling, Simulation, Visualization and Applications (FSPMA)*: 194–203.

Surový P, Ribeiro N, Pereira JS. 2011. Observations on 3-dimensional crown growth of stone pine. *Agroforestry systems* 82 (2): 105–110.

Taugourdeau O, Barczi JF. 2013. Simulating the effect of extreme climatic events on tree architecture with a minimal FSPM. *Proceedings of the 7th International Conference on Functional-Structural Plant Models*: 288–290.

Vaccari S, Calders K, Herold M, Bartholomeus H, Van Leeuwen M. 2013. Terrestrial laser scanning for 3D forest modeling and reflectance simulation through radiative transfer. *Proceedings SilviLaser 2013, 13th International Conference on LiDAR Applications for Assessing Forest Ecosystems*: 18–25.

Vaillant J, Grechi I, Normand F, Boudon F. 2022. Towards virtual modelling environments for functional–structural plant models based on Jupyter notebooks: application to the modelling of mango tree growth and development. *in silico Plants* 4 (1): diab040.Van

Leeuwen M, Nieuwenhuis M. 2010. Retrieval of forest structural parameters using LiDAR remote sensing. *European Journal of Forest Research* 129 (4): 749–770.

Vos J, Evers JB, Buck-Sorlin GH, Andrieu B, Chelle M, De Visser PH. 2010. Functional–structural plant modelling: A new versatile tool in crop science. *Journal of experimental Botany* 61 (8): 2101–2115.

Wang D, Momo Takoudjou S, Casella E. 2020. LeWoS: A universal leaf-wood classification method to facilitate the 3D modelling of large tropical trees using terrestrial LiDAR. *Methods in Ecology and Evolution* 11 (3): 376–389.

Wang F, Kang M, Lu Q, et al. (2011). A stochastic model of tree architecture and biomass partitioning: application to Mongolian Scots pines. *Annals of Botany* 107 (5): 781–792.

Wang Y, Lehtomäki M, Liang X, et al. 2019. Is field-measured tree height as reliable as believed – A comparison study of tree height estimates from field measurement, airborne laser scanning and terrestrial laser scanning in a boreal forest. *ISPRS Journal of Photogrammetry and Remote Sensing* 147: 132–145.

Wilkes P, Lau A, Disney M, et al. 2017. Data acquisition considerations for terrestrial laser scanning of forest plots. *Remote Sensing of Environment* 196: 140–153.

Wilkes P, Disney M, Armston J, et al. 2023. TLS2trees: a scalable tree segmentation pipeline for TLS data. *Methods in Ecology and Evolution* 14(12): 3083-3099.

Yun T, An F, Li W, Sun Y, Cao L, Xue L. 2016. A novel approach for retrieving tree leaf area from ground-based LiDAR. *Remote Sensing* 8 (11): 942.

Zhang C, Yang G, Jiang Y, et al. 2020. Apple tree branch information extraction from terrestrial laser scanning and backpack-lidar. *Remote Sensing* 12 (21): 3592.

Zhang Y, Jia WW. 2021. Extraction of branch factors and model construction for Larix plantation using terrestrial laser scanning (TLS). Ying Yong Sheng Tai Xue Bao 32 (7): 2505–2513.

Zheng G, Moskal LM, Kim, SH. 2012. Retrieval of effective leaf area index in heterogeneous forests with terrestrial laser scanning. *IEEE Transactions on Geoscience and Remote Sensing* 51 (2): 777–786.

Zolkos S, Goetz S, Dubayah R. 2013. A meta-analysis of terrestrial aboveground biomass estimation using lidar remote sensing. *Remote Sensing of Environment* 128: 289–298.

Figure legends with alt text

Figure 1 Examples of manual corrections made in the points clouds to adjust for errors in the LiDAR data and the output of the classification algorithm LeWoS (D. Wang et al., 2020). Woody components are shown in purple and soft components in green.

Figure 1 (alt text) Graphs comparing (before and after) manual corrections done to branches to adjust for errors in the pointcloud data.

Figure 2 Flowchart with the results from the systematic literature search. Figure derived from the PRISMA method (Moher et al., 2009).

Figure 2 (alt text) Flowchart depicting results of systematic literature search (derived from PRISMA method).

Figure 3 Preprocessing steps performed to acquire LiDAR-derived tree parameters. A.) An individual segmented tree PCD was used as input. B.) The tree PCD was classified using the LeWoS algorithm (D. Wang et al., 2020) into woody components (brown) and soft components (green). C.) The soft components were discarded. D.) The PCD was manually corrected, removing wrongly classified soft components and noise in the PCD. E.) A QSM (Raumonen et al., 2013) was fitted for the manually corrected PCD.

Figure 3 (alt text) Graphs showing the different preprocessing steps to derive LiDAR derived tree parameters.

Figure 4 QSM outputs comparison of one tropical tree (treeID: 80_14) between the direct output of the classification algorithm and PCD with additional manual corrections. A.) Visual comparison of the woody component PCD used as QSM input. B.) Visual comparison of the QSM outputs. C.) Histogram with the difference of the branch order count QSM output D.) Histogram and normal distribution function of branch angle QSM output.

Figure 4 (alt text) Graphs comparing (before and after manual corrections) between tree pointclouds and QSM models on top and on the bottom part the distribution of the branch order count and branch angle (before and after manual correction).

Figure 5 Distributions of the LiDAR-derived FSP model inputs. Species abbreviations and number of trees: G = Greenheart (10), K = Kabukalli (5), M = Mora (4), MB = Morabukea (9), WS = Wallaba soft (5), W= Wamara (4), SP = Scots pine (10).

Figure 5 (alt text) Graphs depicting different distributions of branch angle and internode length per first and second branch order, illustrated with boxplots for 7 different tree species.

Figure 6 Accuracy of LiDAR-derived parameters for tropical trees and Scots pine trees. Field observations of height and diameter at breast height (DBH) plotted against TLS-derived estimations. Additionally, measurements from branch angle and internode length which were measured directly from the PCD are plotted against the TLS-derived estimations. The black dotted line is the 1:1 line and the red line represents the regression line. Reported R^2, RMSE and RMSE(%) of each LiDAR-derived parameter can be found in Table 4.

Figure 6 (alt text) Graphs showing accuracy between measured and LiDAR derived parameters for several tree parameters for tropical trees and Scots pine trees.

Figure 7 Tropical tree FSP model variable output for different LiDAR-derived parameter inputs. The median of the LiDAR-derived branch angle was used, as well as the 25th percentile (min), 75th percentile (max) and a normal distribution function. The average was taken of the variable outputs for the species-specific LiDAR-derived parameters. The default input settings were used to compare the outputs of the LiDAR-derived parameters.

Figure 7 (alt text) Graphs showing different model variable outputs for different LiDAR derived parameter inputs as a tangent function for tropical trees FSP models

Figure 8 Visual differences over time of the outputs of the tropical tree FSP model with different LiDAR-derived parameter inputs. Ten TLS scans of Greenheart trees (Chlorocardium rodiei) were used for the LiDAR-derived inputs. The median of the LiDAR-derived branch angle was used, as well as the 25th percentile (min), 75th percentile (max) and a normal distribution function.

Figure 8 (alt text) Figures showing visual difference among tropical tree models per LiDAR derived parameter input scale to the tree lifetime (from 10 years to 200 years)

Figure 9 Scots pine FSP model variable output for different LiDAR-derived parameter inputs. The median of the LiDAR-derived branch angle was used, as well as the 25th percentile (min), 75th percentile (max) and a normal distribution function. The default input settings were used to compare the outputs of the LiDAR-derived parameters.

Figure 9 (alt text) Graphs showing different model variable outputs for different LiDAR derived parameter inputs as a tangent function for Scots trees FSP models

Figure 10 Visual differences over time of the outputs of the Scots pine FSP model with different LiDAR-derived parameter inputs. The median of the LiDAR-derived branch angle was used, as well as the 25th percentile (min), 75th percentile (max) and a normal distribution function. An additional closeup of the last growth year was added to highlight the structural differences.

Figure 10 (alt text) Figures showing visual difference among tropical Scots pine models per LiDAR derived parameter input scale to the tree lifetime (from 10 years to 200 years).

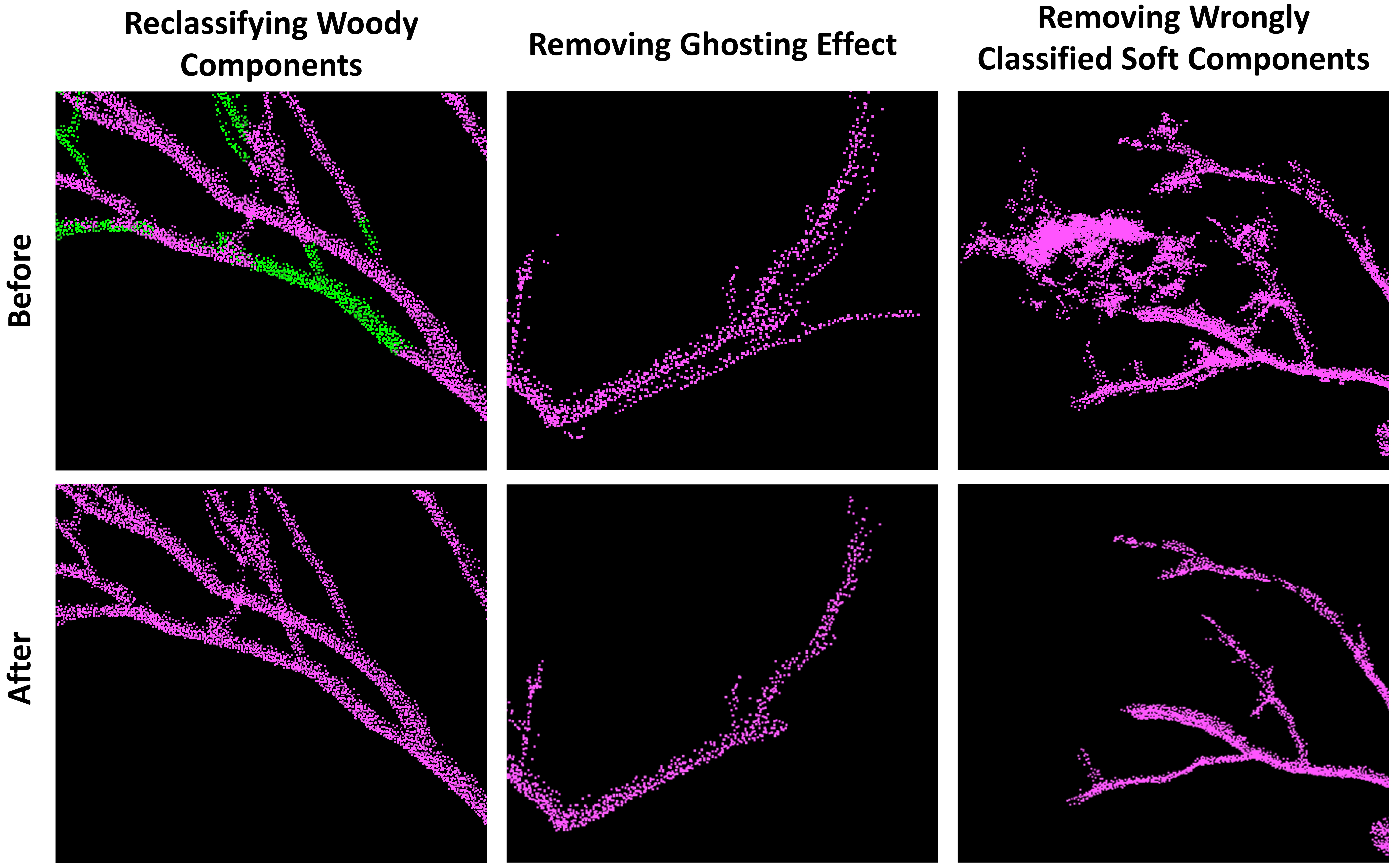
Reclassifying Woody Components
Removing Ghosting Effect
Removing Wrongly Classified Soft Components
Before
After

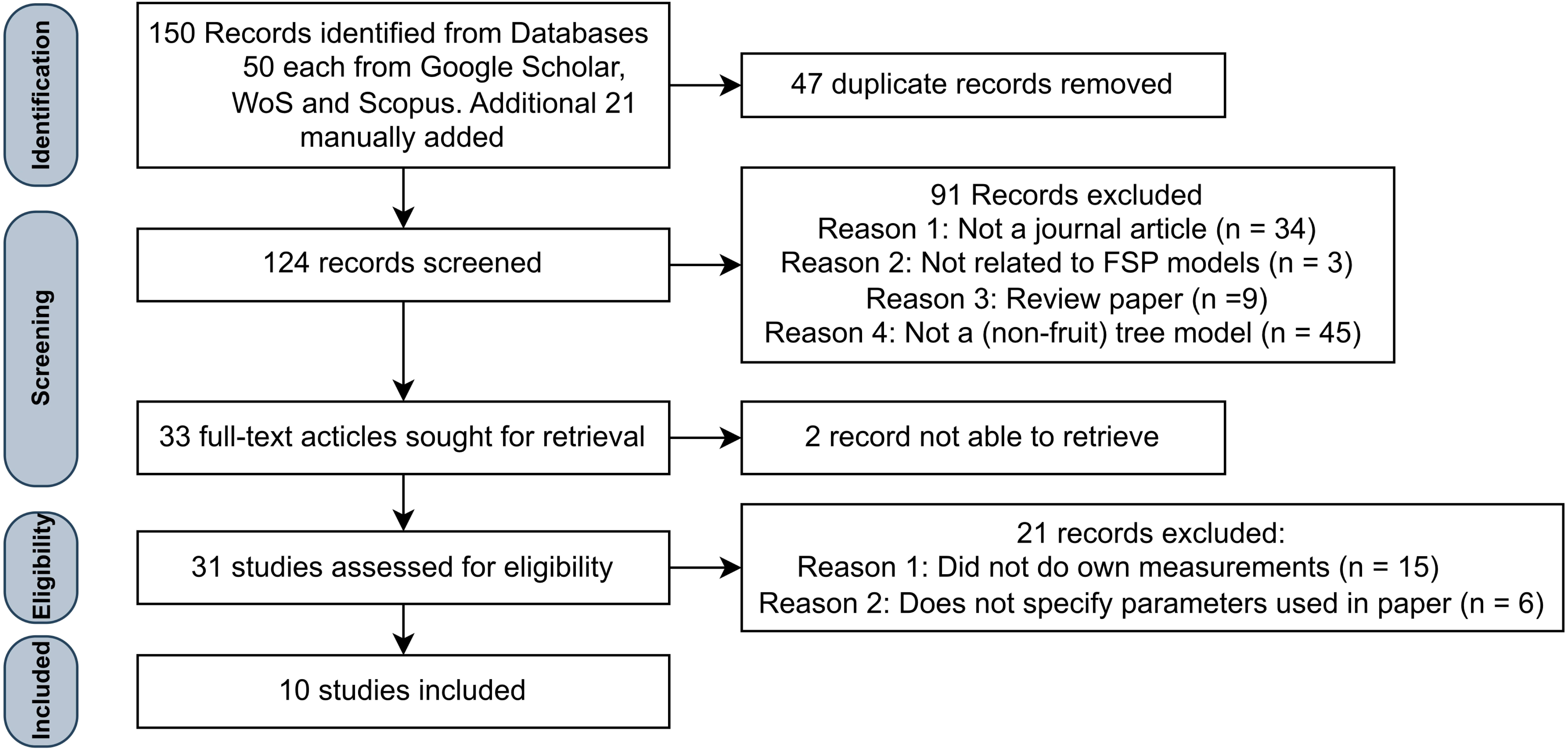
Identification
Screening
Eligibility
Included
150 Records identified from Databases 50 each from Google Scholar, WoS and Scopus. Additional 21 manually added
47 duplicate records removed
124 records screened
91 Records excluded
Reason 1: Not a journal article (n = 34)
Reason 2: Not related to FSP models (n = 3)
Reason 3: Review paper (n =9)
Reason 4: Not a (non-fruit) tree model (n = 45)
33 full-text acticles sought for retrieval
2 record not able to retrieve
31 studies assessed for eligibility
21 records excluded:
Reason 1: Did not do own measurements (n = 15)
Reason 2: Does not specify parameters used in paper (n = 6)
10 studies included

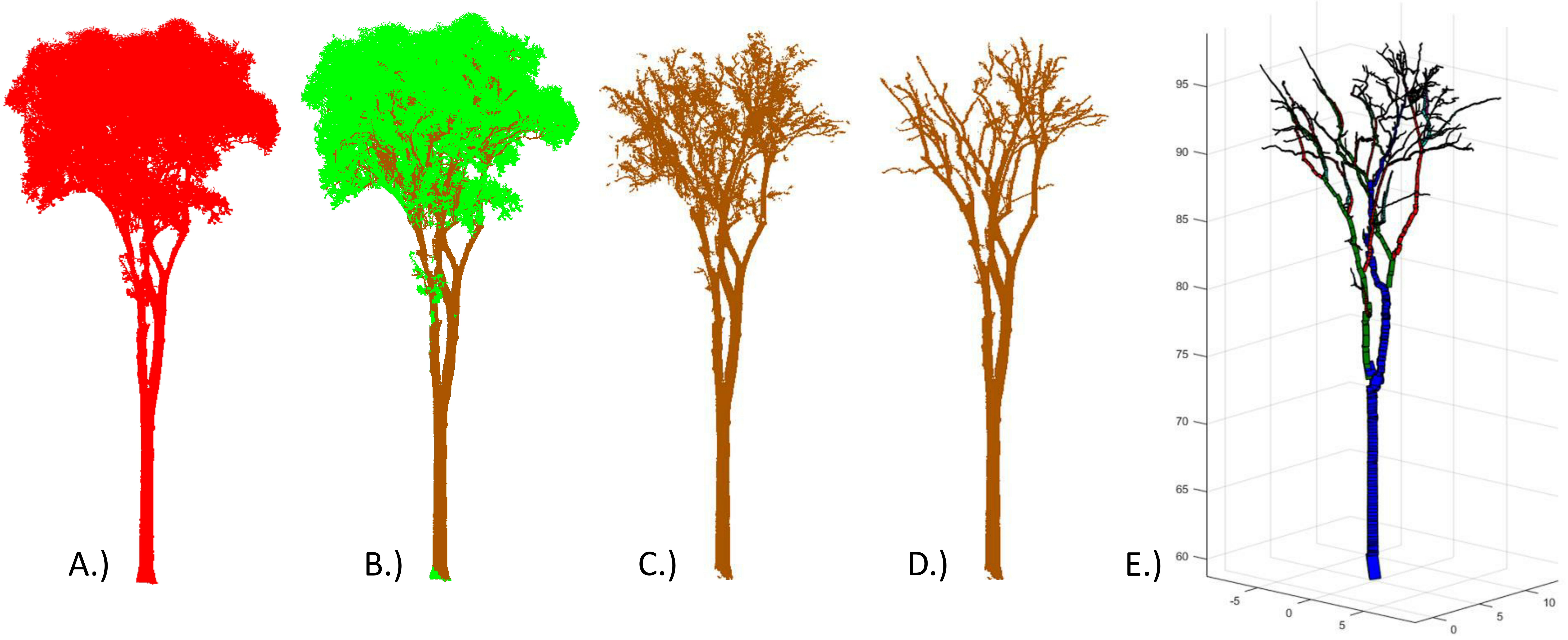
A.)
B.)
C.)
D.)
E.)
95
90
85
80
75
70
65
60
-5
0
5
0
5
10

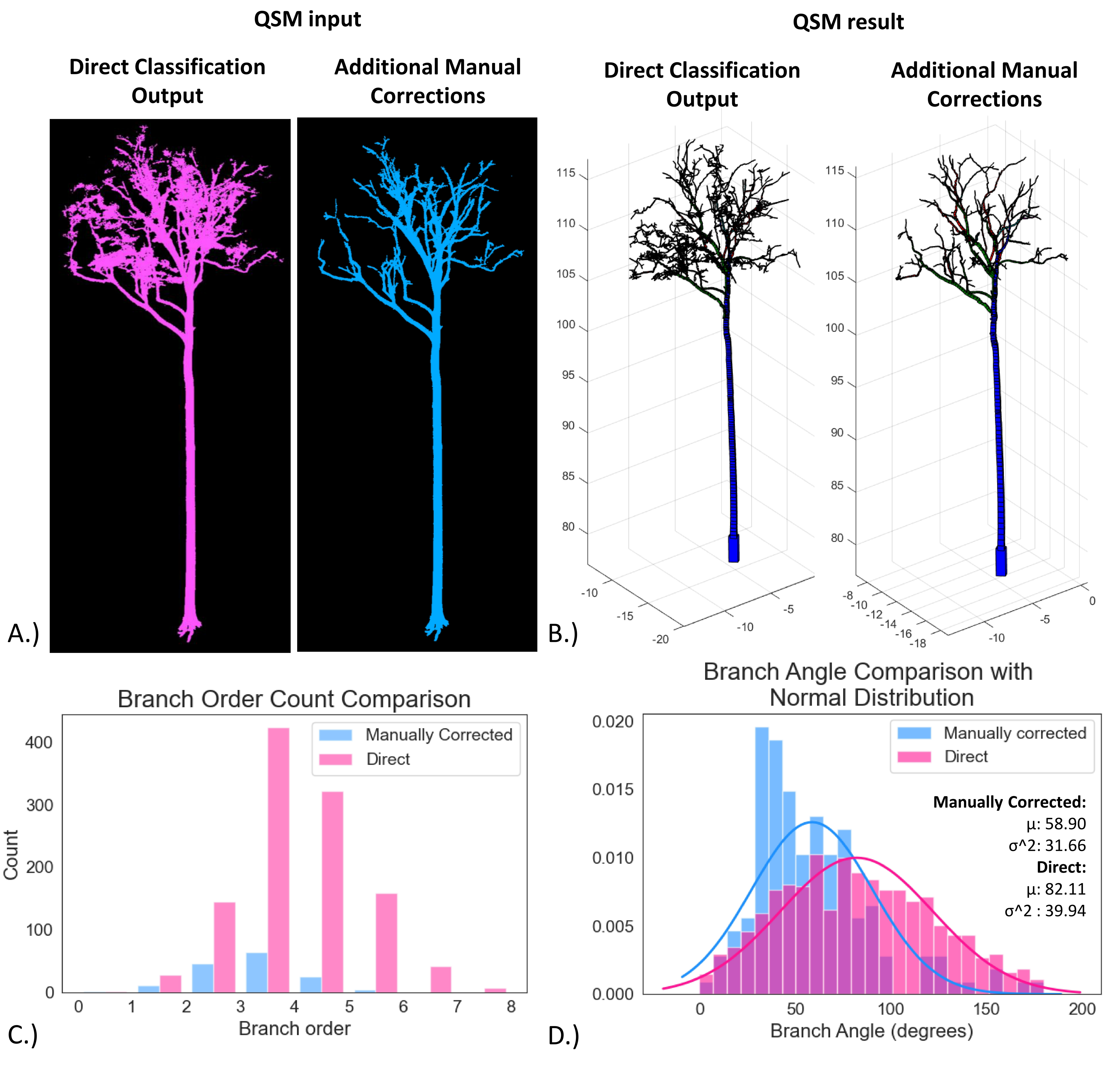

QSM input
QSM result
Direct Classification Output
Additional Manual Corrections
Direct Classification Output
Additional Manual Corrections
A.)
B.)
Branch Order Count Comparison
Manually Corrected
Direct
Count
Branch order
C.)
Branch Angle Comparison with Normal Distribution
Manually corrected
Direct
Manually Corrected:
μ: 58.90
σ^2: 31.66
Direct:
μ: 82.11
σ^2 : 39.94
Branch Angle (degrees)
D.)

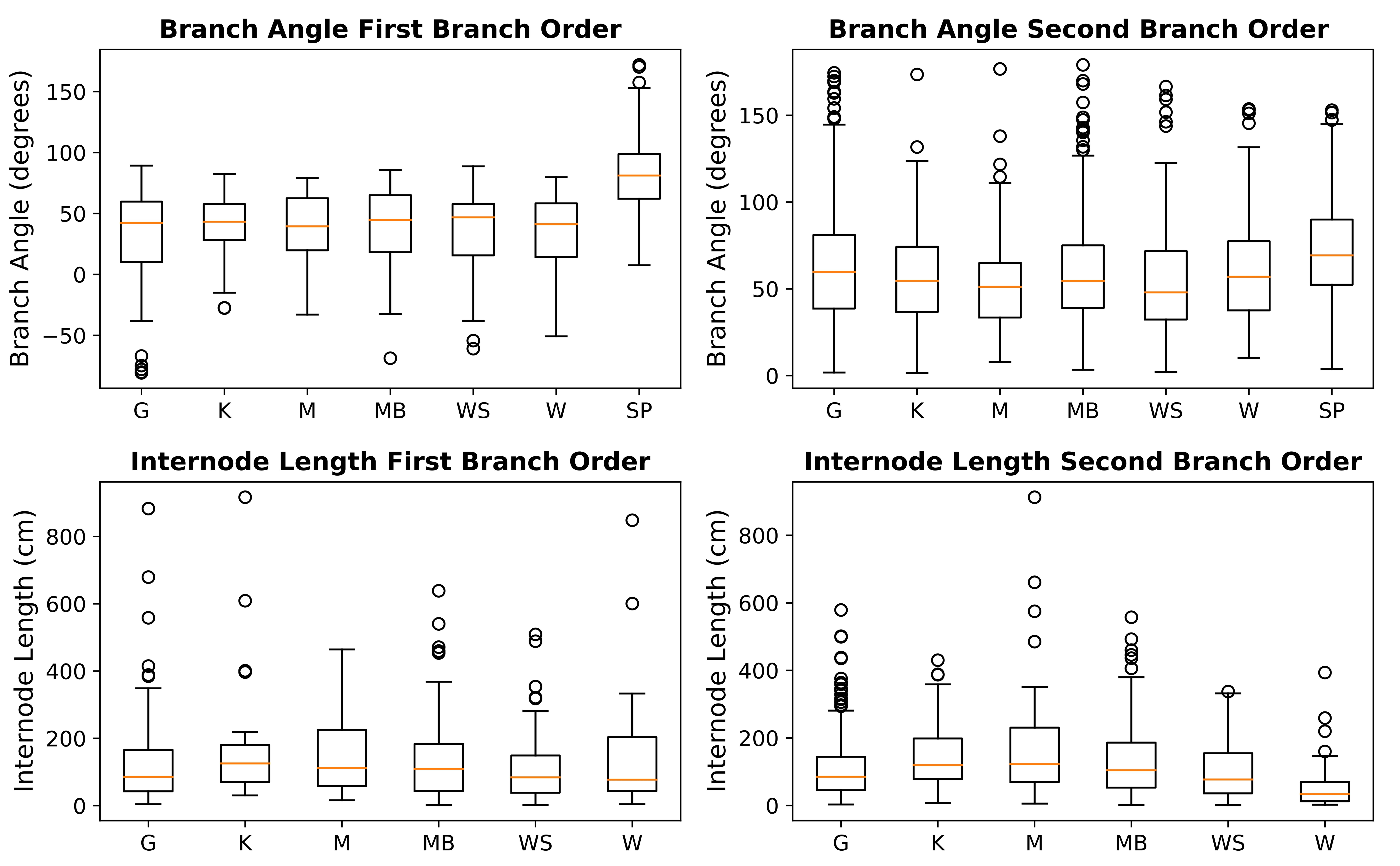

Branch Angle First Branch Order
Branch Angle (degrees)
150
100
50
0
−50
G
K
M
MB
WS
W
SP
Branch Angle Second Branch Order
Branch Angle (degrees)
150
100
50
0
G
K
M
MB
WS
W
SP
Internode Length First Branch Order
Internode Length (cm)
800
600
400
200
0
G
K
M
MB
WS
W
Internode Length Second Branch Order
Internode Length (cm)
800
600
400
200
0
G
K
M
MB
WS
W

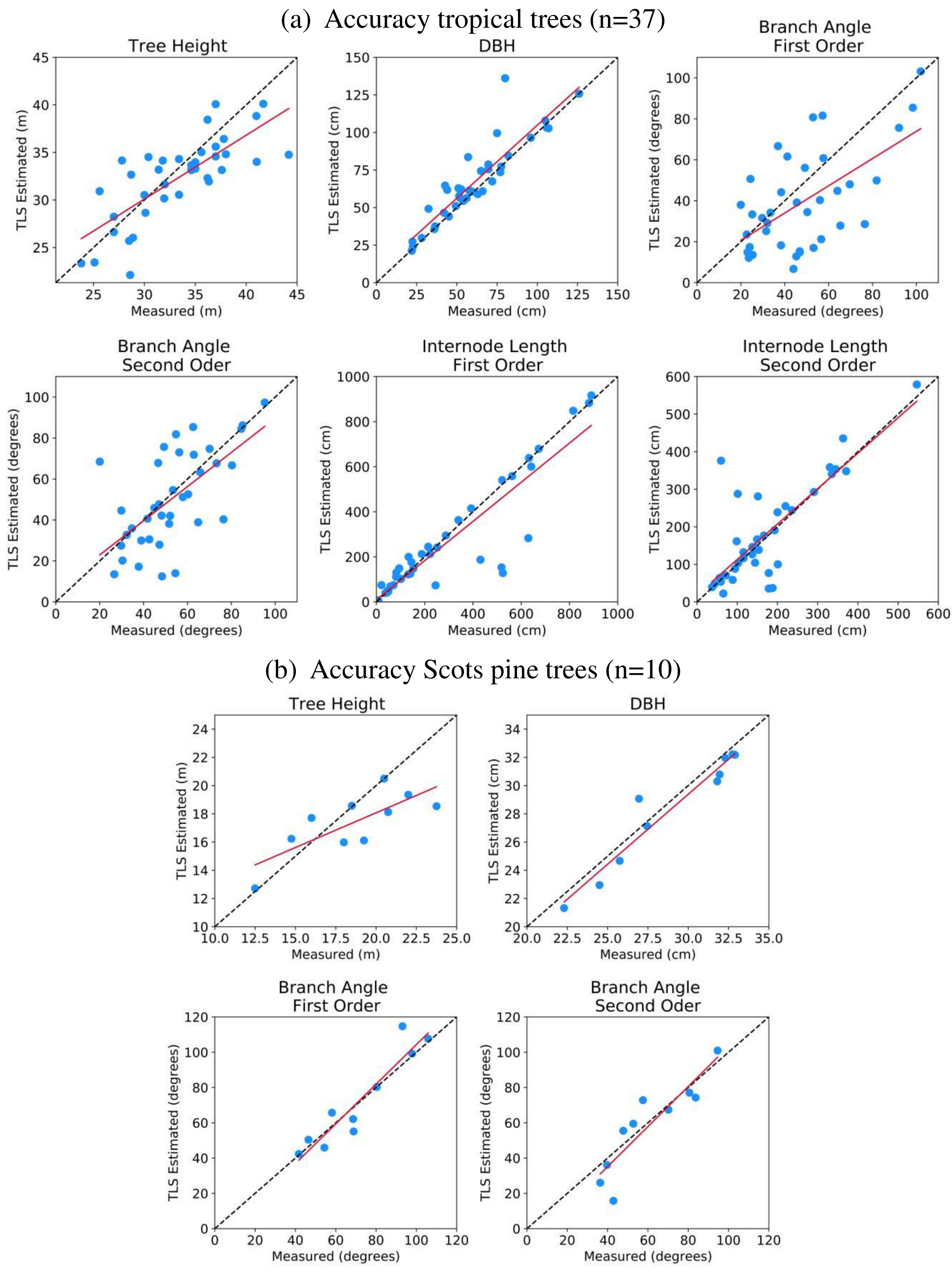
(a) Accuracy tropical trees (n=37)
Tree Height
DBH
Branch Angle First Order
Branch Angle Second Oder
Internode Length First Order
Internode Length Second Order
TLS Estimated (m)
Measured (m)
TLS Estimated (cm)
Measured (cm)
TLS Estimated (degrees)
Measured (degrees)
(b) Accuracy Scots pine trees (n=10)
Tree Height
DBH
Branch Angle First Order
Branch Angle Second Oder

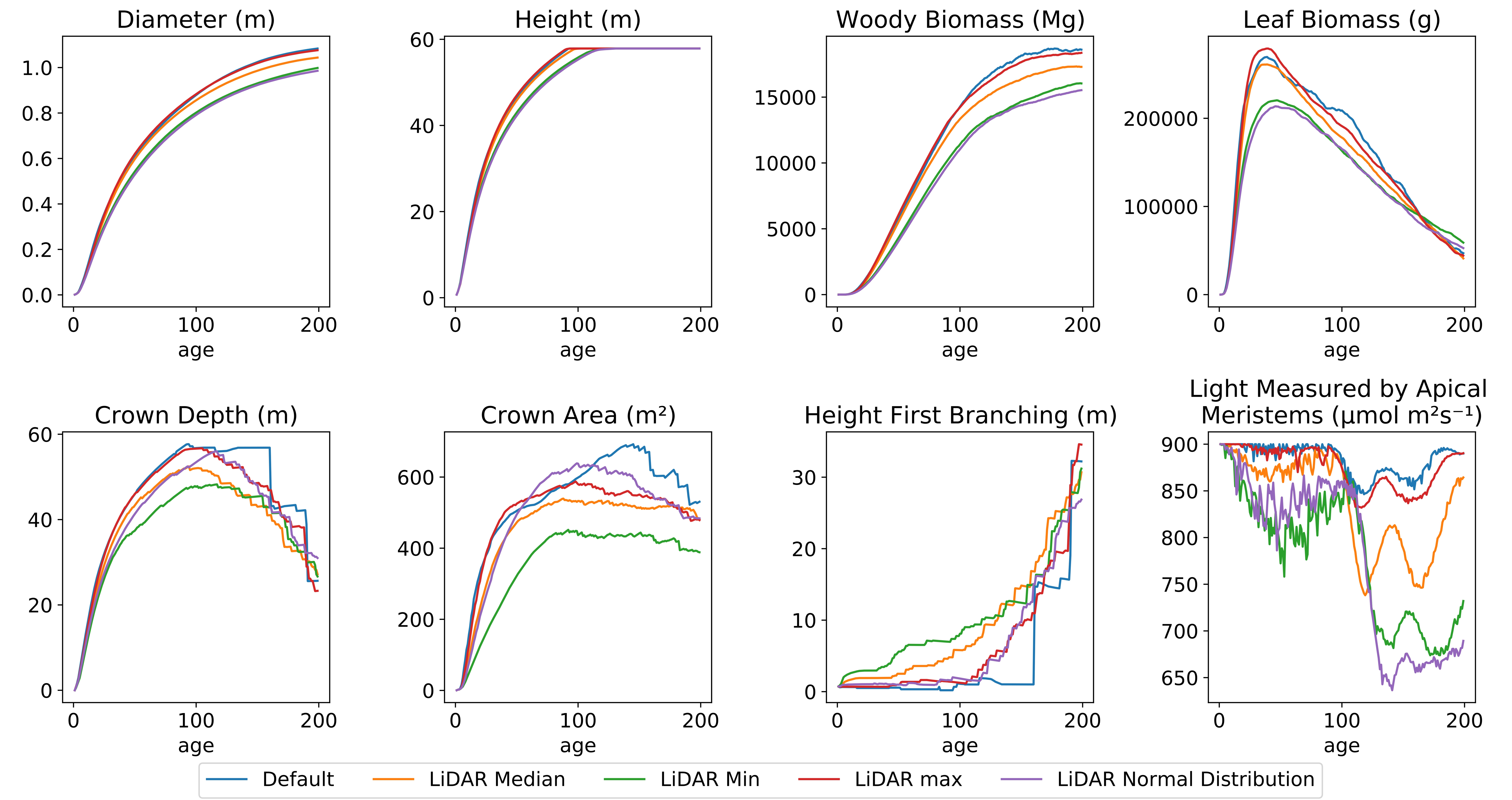
Diameter (m)
Height (m)
Woody Biomass (Mg)
Leaf Biomass (g)
Crown Depth (m)
Crown Area (m²)
Height First Branching (m)
Light Measured by Apical Meristems (µmol m²s⁻¹)
age
Default
LiDAR Median
LiDAR Min
LiDAR max
LiDAR Normal Distribution

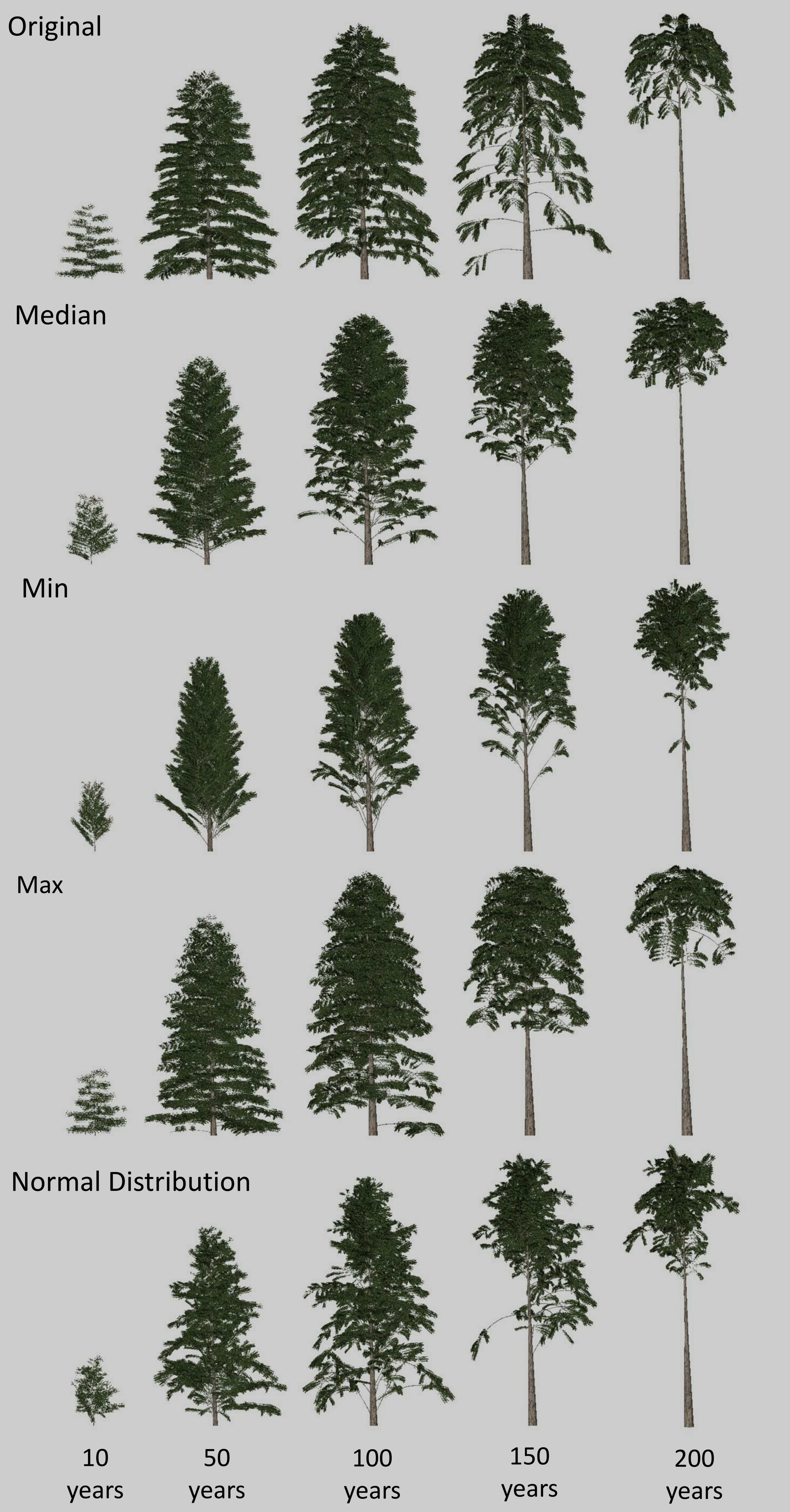
Original
Median
Min
Max
Normal Distribution
10 years
50 years
100 years
150 years
200 years

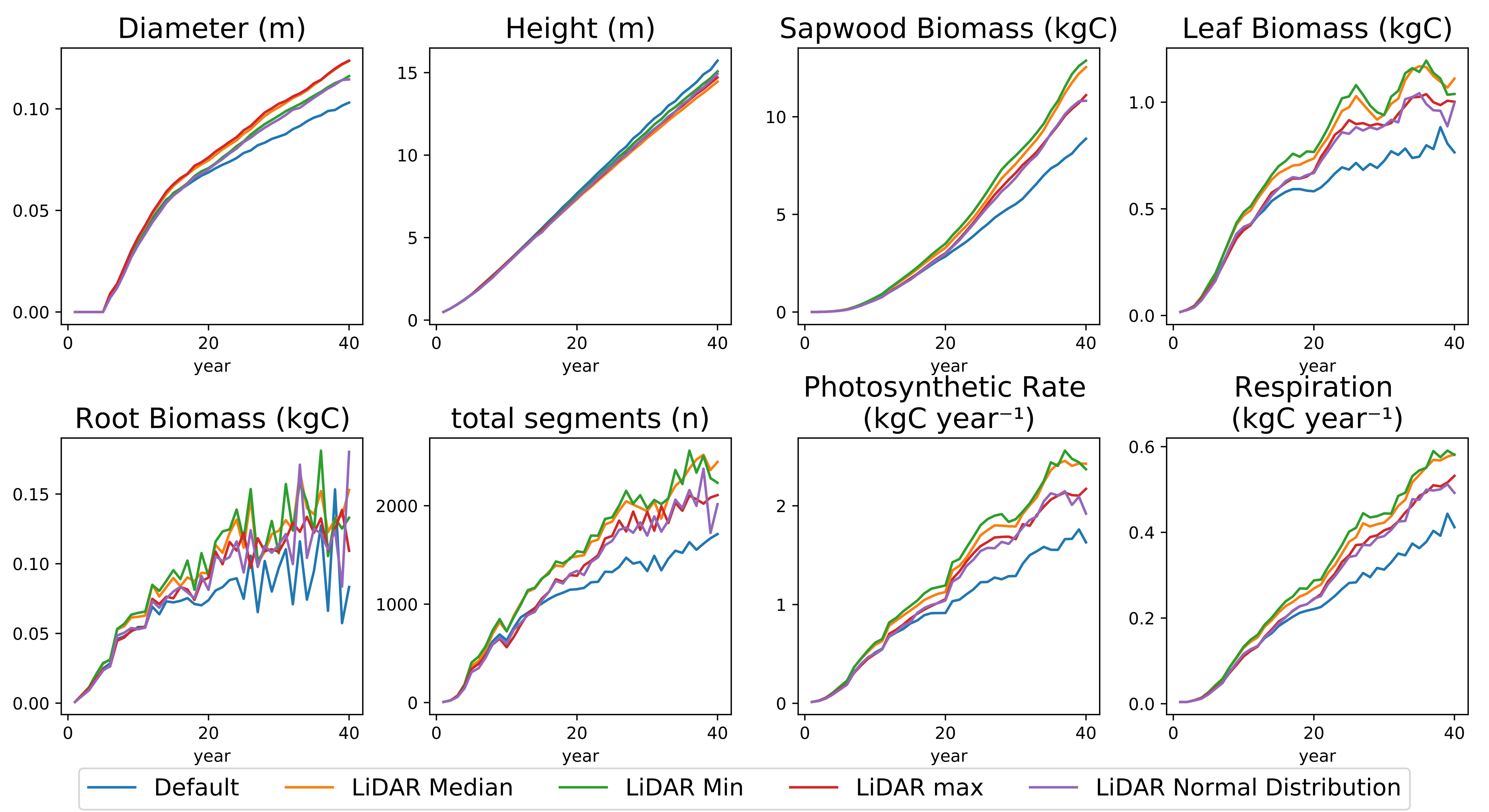

Diameter (m)
Height (m)
Sapwood Biomass (kgC)
Leaf Biomass (kgC)
Root Biomass (kgC)
total segments (n)
Photosynthetic Rate (kgC year⁻¹)
Respiration (kgC year⁻¹)
year
Default
LiDAR Median
LiDAR Min
LiDAR max
LiDAR Normal Distribution

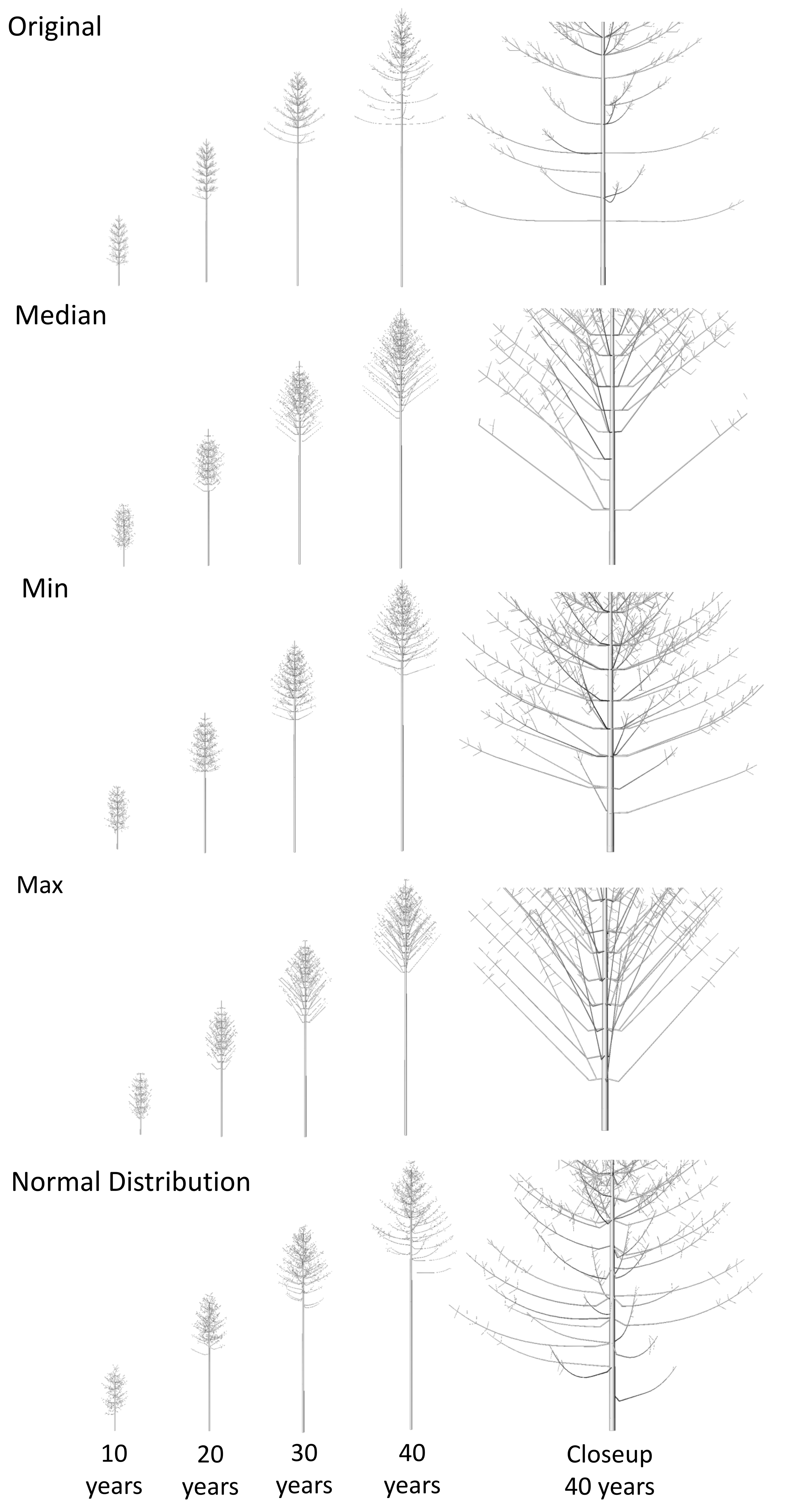
Original
Median
Min
Max
Normal Distribution
10 years
20 years
30 years
40 years
Closeup 40 years

Table 1: Specifics of the LiDAR data used for deriving structural tree parameters.

Plot Location	LiDAR Scanner	Species and Number of Trees	Field Measurements Height (m)	Field Measurements DBH (cm)
Loobos, The Netherlands	TLS	Scots Pine (*Pinus sylvestris*) = 10	Range: 12.5–23.8, Average: 18.6	Range: 22.3–32.9, Average: 28.9
East Berbice-Corentyne Region, Guyana	TLS	Greenheart(*Chlorocardium rodiei*) = 10,<br>Kabukalli (*Goupia glabra*) = 5,<br>Mora (*Mora excelsa*) = 4,<br>Morabukea (*Mora gonggrijpii*) = 9,<br>Wallaba soft (*Eperua falcata*) = 5,<br>Wamara (*Swartzia leiocalycina*) = 4	Range: 23.8–44.2 Average: 33.3	Range: 22.0–126.0 Average: 60.9

*Table 2: Overview of FSP model parameters found through the literature review and possibilities of LiDAR to derive these. Parameters are grouped by level of detail and literature was searched for each parameter. Deriving the parameter with LiDAR was deemed possible if literature was found that had researched this. Used LiDAR scanner and accuracies are summarized for each paper. Finally, the documentation of the QSM (Raumonen et al., 2013) was examined to identify parameters that are derivable through the cylinder outputs. TLS = Terrestrial LiDAR Scanner. ALS = Airborne LiDAR Scanner. * = indicates that it has the potentiality to be derived from the QSM cylinder information*

**Level of Detail**	**FSP Model Parameters Needs**	**LiDAR Scanner**				**QSM**
		**Possible**	**Paper**	**LiDAR method**	**Accuracy**	**Possible**
Neighbourhood	Relative positions to closest neighbours	✓	Ritter et al. (2017)	TLS	97.2% correct	-
	Number of competitors	✓	Ritter et al. (2017)	TLS	-	-
	Crown radius of surrounding neighbours	✓	(see crown diameter)	-	-	-
Individual Tree	Species	✓	Åkerblom et al. (2017)	TLS	93% correct	-
	Age	✓	Rizeei et al. (2018)	ALS	84.91% correct	-
	Height	✓	Calders, Newnham, et al. (2015)	TLS	(R^2=0.94, RMSE=1.28 m)	✓
	DBH	✓	Calders, Newnham, et al. (2015)	TLS	(R^2=0.97, RMSE=2.39 cm)	✓
	Crown diameter	✓	Fernández-Sarría et al. (2019)	TLS	(R^2=0.92, RMSE=0.29 m)	✓
	Total woody biomass	✓	Calders, Newnham, et al. (2015)	TLS	RMSE%=9.7%	✓
	Total foliage biomass	✓	Stovall et al. (2017)	TLS	(R^2=0.63, RMSE=5.2 kg)	-
	Crown base height	✓	Popescu and Zhao (2008)	ALS	(R^2=0.80, RMSE=2.03 m)	✓

	Number of branches	✓	C. Zhang et al. (2020)	TLS	(RMSE%: 1st order=12%, 2nd=9.67%, 3rd=23.81%, 4th=164.17%)	✓
Whorls	Number of whorls	✓	Pyörälä et al. (2018)	TLS	69.9% detected	-
	Number of branches per whorl	✓	Klemmt et al. (2010)	TLS	-	-
	Stem diameter above and below the whorl	-	-	-	-	-
	Height of whorl	✓	Klemmt et al. (2010)	TLS	(R2: 1st whorl=0.92, 2nd whorl=0.88, 3rd whorl=0.67)	-
Branches	Branch location	-	-	-	-	✓
	Branch order	✓	Lau et al. (2018)	TLS	99% correct	✓
	Branch age	-	-	-	-	-
	Branch diameter	✓	Lau et al. (2018)	TLS	(10-20 cm=40% overest., 20-60 cm=8% underest., 60 cm>6% underest.)	*
	Branch base diameter	✓	Bucksch and Fleck (2011)	TLS	R^2=0.98	✓
	Branch diameter below and above branching point	-	-	-	-	*
	Branch angle	✓	Pyörälä et al. (2018)	TLS	RMSE=7.76°	✓
	Branch bending angle	-	-	-	-	*
	Branch azimuth	-	-	-	-	✓
	Branch length	✓	Lau et al. (2018)	TLS	(50 cm <=20% underest., 50 cm>=1%	✓

					overerest.)	
	Chord length	✓	Y. Zhang and Jia (2021)	TLS	90.6%	*
	Horizontal extent	-	-	-	-	*
	Height of insertion points	✓	Delagrange and Rochon (2011)	TLS	(Mean dev.=1.8 %, Mean abs. error=3 cm)	*
	Total shoots number	✓	Pallas et al. (2020)	TLS	(R^2=0.81, nRMSE=1.63)	-
	Total shoots length	✓	Pallas et al. (2020)	TLS	(R^2=0.97, nRMSE=0.2 m)	-
	Branch mortality	-	-	-	-	-
	Ramification number	-	-	-	-	*
Internode	Internode location along the stem	-	-	-	-	*
	Internode length	✓	Saeed and Li (2021)	TLS	(RMSE=1.04 cm, R^2=0.194)	*
	Internode diameter	-	-	-	-	*
	Total number of internodes	-	-	-	-	*
	Number of Phytomers	-	-	-	-	-
	Internode fresh/dry biomass	-	-	-	-	*
	Needles fresh/dry biomass per pythomer	-	-	-	-	-
Foliage	Specific leaf area	-	-	-	-	-
	Number of leaves per shoot	-	-	-	-	-
	Needle length	-	-	-	-	-
	Needle	-	-	-	-	-

	diameter					
	Geometry of foliage clumps	✓	Ma et al. (2017)	TLS	-	-
	Leaf stalk	-	-	-	-	-
	Leaf height	-	-	-	-	-
	Leaf width	-	-	-	-	-
	Leaf orientation	✓	Stovall et al. (2021).	TLS	(RMSE: 12.7°-18.2°)	-
	Surface area leave	✓	Yun et al. (2016)	TLS	-	-

Table 3: Selection of QSM outputs (Raumonen et al., 2013) of the tropical tree and Scots pine PCD. Differences of outputs are calculated between inputs of direct outputs of the classification algorithm LeWoS (D. Wang et al., 2020) and the PCD with additional manual corrections

		**DBH (cm)**	**Tree height (m)**	**Total volume (L)**	**Trunk volume (L)**	**Branch volume (L)**	**Trunk length (m)**	**Branch length (m)**	**Number of branches**	**Max branch order**	**Total area (m2)**
Tropical trees n=5	Direct	83.2	32.0	10547.8	8400.2	2147.9	31.6	476.5	667.0	7.6	144.3
	Manually corrected	82.9	31.7	10135.9	8481.2	1655.0	32.0	170.2	105.8	5.4	97.3
	Difference	0%	-1%	-8%	1%	-28%	1%	-64%	-84%	-29%	-34%
Scots pine n=10	Direct	28.2	14.3	924.0	537.6	386.4	13.7	176.9	389.0	5.9	37.3
	Manually corrected	28.3	14.4	651.8	548.6	103.2	14.2	41.8	77.9	3.5	16.3
	Difference	0%	0%	-29%	2%	-73%	3%	-76%	-80%	-41%	-56%

Table 4: Accuracy metrics of the QSM outputs for both the tropical trees and Scots pines. LiDAR-derived height and DBH are compared to field observations. The branch angle and internode length are compared to measurements in the PCD.

	**Tropical trees**			**Scots Pine**		
	**RMSE**	**RMSE%**	**R^2**	**RMSE**	**RMSE%**	**R^2**
**Height (m)**	3.40	10.22	0.58	2.45	13.19	0.59
**DBH (cm)**	12.96	21.29	0.81	1.17	4.05	0.93
**Branch Angle 1st order (°)**	22.19	46.11	0.36	9.28	12.97	0.88
**Branch Angle 2nd order (°)**	18.1	34.32	0.41	11.59	19.11	0.8
**Internode Length 1st order (cm)**	118.62	38.98	0.81	-	-	-
**Internode Length 2nd order (cm)**	79.74	45.60	0.64	-	-	-

Table 5: The LiDAR-derived branch angle inputs that were used for the tropical tree and Scots pine FSP Models. Tropical tree LiDAR-derived branch angle of the first-order is relative to the horizontal plane. Scots pine and second-order tropical tree branch angle are calculated by taking the angle between trunk and first-order branches (first-order branch angle) and first and second branches (second-order branch angle).

**Species**	**Branch Angle 1ste Order (degrees)**			**Branch Angle 2nd Order (degrees)**		
	**Median**	**25th- 75th Percentile Range**	**Normal Distribution**	**Median**	**25th-75th Percentile Range**	**Normal Distribution**
**Greenheart**	42	60-10	μ = 32, σ = 38	60	39-81	μ = 64, σ = 35
**Kabukalli**	43	58-28	μ = 39, σ = 25	55	37-74	μ = 57, σ = 31
**Mora**	39	62-20	μ = 38, σ = 30	51	33-65	μ=53, σ = 29
**Morabukea**	45	65-18	μ = 39, σ = 31	55	39-75	μ = 61, σ = 33
**Wallaba soft**	47	58-16	μ = 34, σ = 35	48	32-72	μ = 56, σ = 34
**Wamara**	41	58-14	μ = 38, σ = 30	57	38-77	μ = 65, σ = 39
**Scots Pine**	81	62-99	μ = 82, σ = 29	69	52-90	μ = 72, σ = 31
